# Improving the efficiency of 4A-zeolite synthesized from kaolin by amine functionalization for CO_2_ capture

**DOI:** 10.1038/s41598-023-39859-z

**Published:** 2023-08-02

**Authors:** Fatemeh Bahmanzadegan, Mahyar Ashourzadeh Pordsari, Ahad Ghaemi

**Affiliations:** School of Chemical, Petroleum and Gas Engineering, Iran University of Science and Technology, PO Box: 16846-13114, Tehran, Iran

**Keywords:** Environmental chemistry, Chemistry, Chemical engineering

## Abstract

This study focuses on optimizing the CO_2_ adsorption capacity of 4A-zeolite synthesized from kaolin by employing structural modifications through impregnation with tetraethylenepentamine (TEPA) and diethanolamine (DEA). Various analytical techniques were utilized to evaluate the effectiveness of these modifications. Design expert software and response surface methodology (RSM) was employed for data analysis and operational variable optimization, leading to improved CO_2_ adsorption performance of the modified zeolites. The adsorption capacity of the modified zeolites was assessed under different temperatures, pressures, and amine concentrations using a test device. The optimal adsorption capacity of 4A-DEA adsorbent is found to be 579.468 mg/g, with the optimal operational variables including a temperature of 25.270 °C, pressure of 8.870 bar, and amine concentration of 11.112 wt%. The analysis shows that the adsorption process involves both physisorption and chemisorption, and the best kinetic model is the fractional-factor model.

## Introduction

Rising CO_2_ levels in the atmosphere are a critical concern for global climate change and environmental risks. By 2100, it could increase to 26 billion tons per year. This forecast has consequently emphasized the importance of prioritizing the capture and adsorption of CO_2_ from an environmental perspective^1^. The ongoing release of CO_2_ into the atmosphere has resulted in long-term changes in the global climate, including rising temperatures, sea levels, and more frequent occurrences of extreme weather events. Four primary methods for CO_2_ separation have emerged: absorption, adsorption, cryogenic, and membrane technologies^2,3^. The choice of the suitable CO_2_ capture technique relies on several factors, encompassing the origin of CO_2_, the magnitude of the capture process, the desired level of purity for the captured CO_2_, and the envisioned application of the captured CO_2_^4^. At present, absorption and adsorption represent the prevailing methods employed for CO_2_ capture, whereas cryogenic and membrane technologies are still in their nascent stages of development^5^. Researchers are actively engaged in investigating diverse methodologies to adsorb CO_2_ as a means of mitigating its emissions^6^. Porous materials such as zeolite^7^, silica^8^, MOF^9^, carbon^10^, and polymer^11^ have been used to adsorb CO_2_, each of which has its advantages and disadvantages.

Zeolite is a material with a crystalline structure that can be either naturally occurring or synthesized^12^. It contains aluminosilicate minerals and exhibits a distinctive three-dimensional framework with well-organized pores and channels. Zeolites have crystal structures with a rigid framework that includes pores and channels formed as TO_4_, where T can be silica and aluminum. Aluminum atoms attract the oxygens and produce an excellent site for cation transfer^13^. Cation in the structure of zeolites plays a crucial role in CO_2_ capture because it can attract CO_2_ into the zeolite^6^. Zeolites are promising CO_2_ adsorbents with high surface area, suitable pore size, and excellent thermal and chemical stability^14^. Multiple types of zeolites have undergone thorough investigation to assess their potential in adsorbing CO_2_ gas generated from industrial processes. Zeolite 4A^15^, zeolite 13X^16^, ZK-5^17^, ZSM-5^18^, β-zeolite^19^, and Na-X^20^ are among the zeolite types that have demonstrated potential in applications related to CO_2_ capture. These zeolites possess distinctive pore structures, substantial surface areas, and excellent thermal stability, rendering them highly desirable options for CO_2_ adsorption. Zeolite 13X has demonstrated exceptional selectivity for CO_2_^21^. ZK-5 possesses a distinctive cage-like structure that can be modified to improve its adsorption properties for CO_2_^22^. Similarly, ZSM-5^23^ and β-zeolite^24^ have exhibited significant CO_2_ adsorption capacity in the studies. Additionally, Na-X has shown good stability and regeneration properties^25^. Zeolite 4A is characterized by a substantial concentration of adsorption sites attributable to the presence of aluminum atoms within its framework. These sites exhibit a robust affinity towards CO_2_ molecules, facilitating effective capture and retention of the gas.

There are several methods for synthesizing zeolites, such as hydrothermal synthesis, sol–gel synthesis, microwave-assisted synthesis, and organic template synthesis. The hydrothermal Method is the most commonly used technique for synthesizing zeolite when working with kaolin^26^. Kaolin-based zeolites have high adsorption capacity due to their combination of mesoporous and microporous structures. Its natural abundance makes it a cost-effective option for large-scale applications, and its environmentally friendly properties make it a sustainable choice for CO_2_ capture solutions. The unique characteristics of kaolin-based zeolite allow for customization and tailored modifications, resulting in enhanced CO_2_ capture performance^27,28^.

In recent literature, the modification of zeolites with amine functional groups was studied, and it was demonstrated to enhance their CO_2_ capture capabilities^27,29–34^. Other modifications, such as carbon modification^35^, silica modification^7^, MOF modification^36^, acid treatment^37^ and ion exchange^38^ have also been investigated, showcasing their potential in improving the CO_2_ adsorption performance of zeolites. Structural modifications achieved through various techniques, as demonstrated in relevant research studies^39,40^, can improve the adsorption performance of Zeolite 4A. presents a comprehensive analysis of the advantages and disadvantages of these modifications.

Table 1 presents a comprehensive analysis of the advantages and disadvantages of these modifications.

**Table 1:** Comparison of different methods of zeolite modification.

Modification Route	Advantages	Disadvantages	Ref
Amine functionalization	– Enhanced CO_2_ adsorption capacity – Regenerable and reusable adsorbents – Selective CO_2_ capture	– Potential structural changes – Decreased thermal stability	^27,29–34,40^
Ion exchange	– Simple and cost-effective modification method – Widely applicable to various zeolite types	– Limited control over ion exchange process	^41^
Carbon modification	– Improved adsorbent stability – Enhanced resistance to moisture and temperature – Tunable surface properties	– Limited scalability – Lower CO_2_ adsorption capacity	^35^
MOF modification	– Enhanced stability under high temperature – Increased adsorption capacity and selectivity	– Challenging synthesis and MOF-zeolite integration	^36,42^
Silica modification	– Increased hydrophobicity	– Altered zeolite structure and properties	^7^
Acid Treatment	– Enhanced surface acidity – Improved selectivity	– Potential changes in zeolite structure – Limited control	^37^

The incorporation of amines onto the surface of zeolites can significantly enhance their CO_2_ adsorption capabilities. The interaction between amine-modified zeolites and CO_2_ occurs through chemisorption, wherein a chemical bond forms between the CO_2_ molecule and the amine group on the zeolite surface^43^. As mentioned in presents a comprehensive analysis of the advantages and disadvantages of these modifications.

Table 1, amine-modified zeolites offer multiple advantages, including increased CO_2_ adsorption capacity, selective CO_2_ capture, High regeneration, and low energy consumption^44^. Amine functionalization of zeolites can be achieved through two main methods: grafting and impregnation. The grafting method is a well-established approach that involves attaching amine-containing molecules to the zeolite surface via covalent bonds^45^. This method typically results in higher degrees of functionalization, increased stability of the amine groups, and improved selectivity. However, there may be a reduction in CO_2_ adsorption capacity and difficulties in regeneration due to high pressure^46^. Compared to the grafting method, impregnation is less complex and easier to implement. It allows for a straightforward introduction of functional groups onto the zeolite surface, making it a practical choice for modifying the material^45^. Impregnation method involves immersing the zeolite in a solution containing the desired amine compound to deposit amine-containing molecules onto its surface. After impregnation, the zeolite undergoes washing and drying. Impregnation is a straightforward and versatile approach, but it may result in lower levels of functionalization and less stable amine groups compared to grafting. Amine impregnation involves incorporating amines, such as MEA, DEA, and TEPA, into the pores of a zeolite material^47^. Fashi et al. utilized 2% piperazine to modify zeolite 13X and improve its CO_2_ adsorption capabilities^48^. Babaei et al. examined Na-Y zeolite with a silicon-to-aluminum ratio of 2.5, utilizing varying quantities of amine. When comparing NaY-2-MAE to NaY-2-DEA, they found that the steric barrier is reduced in NaY-2-MAE, leading to increased adsorption. Moreover, five functional groups in TEPA resulted in higher adsorption levels^49^. Ahmad et al. studied the modification of zeolite β by incorporating melamine to enhance its CO_2_ adsorption performance. The modified zeolite demonstrated a significant CO_2_ adsorption capacity of 162.36 mg/g at 298 K and 1 bar, attributable to the increased number of active sites and the improved hydrophobicity of the zeolite surface resulting from the modification^50^. Panda et al. worked on modifying zeolite 4A with different amines such as propylene amine, butyl amine, pentyl amine, isopropyl amine, isobutyl amine, and isopentyl amine. The optimum result of modified-zeolite by butylamine and iso-butylamine was 108.68 and 112.64 mg/g at 298 K and 1 bar^51^. Garshasbi et al. prepared 13 × zeolite and acid modification of Iranian kaolin, which showed an adsorption capacity of 352 mg/g^21^. Thakkar et al. synthesized ZSM-5, Y, and SAPO-34 zeolites using kaolin and modified them using TEPA amine to increase CO_2_ adsorption^27^. Murge et al. synthesized and modified zeolite Y by amine TEPA, the best adsorption performance related to Z-Y-3 at 303 K and 1 bar was 114 mg/g^52^.

Table 2 presents an overview of the studies and experiments on various amines for modifying zeolite structures. The modifications involved alterations in the chemical structure and properties of the zeolites, such as pore size, surface area, and functionality, leading to changes in the adsorption capacity and selectivity. The tabulated results offer insights into the potential of amines as modifying agents for enhancing the adsorption performance of zeolites, as well as the conditions required for achieving optimal results Table 2.

**Table 2:** Review of studies on different modified zeolites with amine for CO_2_ adsorption.

Researcher	Zeolite type	Improving agent	Temperature (K)	Pressure (bar)	CO_2_ capacity (mg/g)	Ref.
Hwang et al.	Zeolite 4A	–	298	15	268.659	^39^
Wang et al.	Zeolite 4A	–	298	45	116.67	^28^
Siriwardane et al.	Zeolite 4A	–	393	1	22.005	^53^
Karimi et al.	Zeolite 4A	–	303	8	224.451	^54^
Mortazavi et al.	Clinoptilolite	MEA	298	4	157.52	^6^
Krachuamram et al.	Zeolite NaX	CTAB	303	1	223.52	^7^
Murge et al.	Zeolite Y	PEI	303	1	113.96	^52^
Nguyen et al.	Zeolite A	APTMS	333	1	101.20	^34^
Nguyen et al.	Zeolite A	TMPED	333	1	61.60	^34^
Panda et al.	Zeolite 4A	B	298	1	108.68	^51^
Panda et al.	Zeolite 4A	IBA	298	1	112.64	^51^
Fashi et al.	Zeolite 13X	–	298	8	193.77	^48^
Fashi et al.	Zeolite 13X	Piperazine	298	8	242.00	^48^
Babaei et al.	Zeolite NaY	–	298, 348	1	82.8, 73.5	^49^
Babaei et al.	Zeolite NaY	DEA	298, 348	1	65.2, 78	^49^
Babaei et al.	Zeolite NaY	TEPA	298, 348	1	60.6, 92.9	^49^
Babaei et al.	Zeolite NaY	MAE	298, 348	1	72.9, 85.4	^49^
Ahmad et al.	Zeolite β	Melamine	298	1	79.94	^50^
Wahon et al.	Natural zeolite	PEI	348	1	14.08	^55^

Researchers frequently utilize the response surface methodology (RSM) as a statistical modeling technique to better understand the behavior of chemical systems and improve their performance. RSM is considered a valuable tool for optimizing chemical processes^56^. Pashaei et al. demonstrated the effectiveness of RSM in optimizing the CO_2_ absorption process into piperazine solutions^5^. Gill et al. used the RSM to evaluate the effect of parameters such as activation temperature and burning degree on CO_2_ absorption capacity^57^. Karimi et al. used the RSM method to model the CO_2_ adsorption capacity by modifying a commercially activated carbon^58^. Khajeh et al. used RSM to optimize the operational conditions, reactor temperature and pressure, and acid concentration for activating the surface and wt% NaOH to raise the adsorption capacity performance^59^. The influence of amine loading on the adsorbent structures derived from kaolin-modified zeolite for CO_2_ capture has received limited attention in previous studies, especially using RSM. This article aims to fill this research gap by investigating the effects of amine loading on these structures and evaluating their capability for CO_2_ adsorption.

In this study, we aim to enhance the CO_2_ adsorption capacity of zeolite synthesized from kaolin. Our focus was on using two specific amines, Tetraethylenepentamine (TEPA) and Diethanolamine (DEA), as modifiers for the zeolite. RSM was utilized to optimize the experiments and operating conditions for the modified zeolites and evaluate their CO_2_ adsorption capability. Furthermore, we analyzed the kinetic and thermodynamic characteristics of CO_2_ capture of adsorbents.

## Material and method

### Materials

Iranian Kaolin was employed to synthesize 4A-zeolite. Sodium hydroxide (NaOH) and methanol were procured from Merck. Tetraethylenepentamine (TEPA) and diethanolamine (DEA), both of analytical grades, were used as amines during the adsorbent synthesis and obtained from Sigma Aldrich.

### 4A-zeolite synthesis

**Figure 1:**
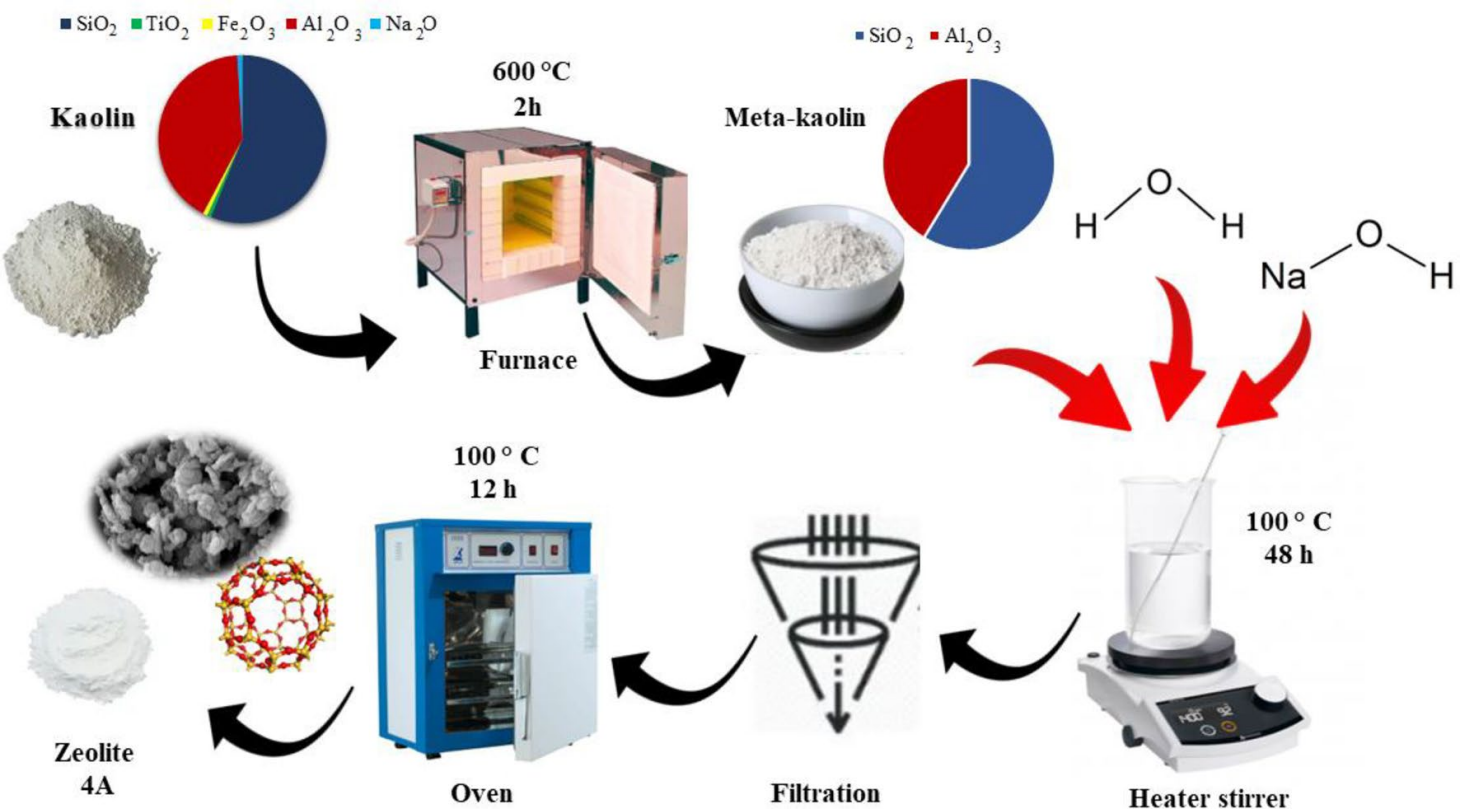
Schematic of 4A-zeolite synthesis.

Zeolite 4A was synthesized using a prescriptive method described in reference^27^. The synthesis process involved the calcination of Iranian Kaolin at a temperature of 600 °C for 2 h, with a heating rate of 5 °C/min. Subsequently, 5 g of metakaolin were immersed in 100 mL of 2M NaOH solution in a round-bottom flask, and treated by stirring under reflux for 48 h at a temperature of 100 °C. The resulting mixture was then washed with deionized water until the pH reached 7, after which it was dried at 100 °C for 12 h. The resulting product was a highly porous 4A-zeolite. The synthesis process is illustrated in Fig. 1.

### Impregnation of 4A-zeolite by DEA and TEPA

**Figure 2:**
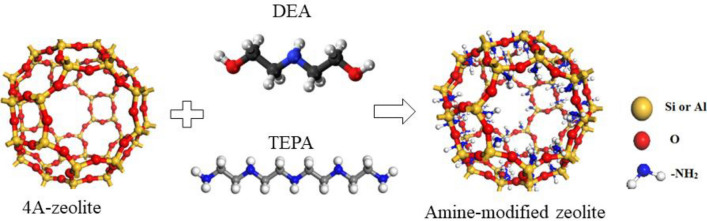
Modification of 4A-Zeolite from kaolin with TEPA and DEA.

The optimal conditions for CO_2_ adsorption experiments, including temperature, pressure, and amine loading, were determined using Design Expert software to create an experimental design. Modified zeolites were then prepared based on the data generated by the software, with an amine loading of 5–25 wt%, to meet the specific requirements of the experimental design. For preparing amine-modified zeolite, we used the wet impregnation route. In this method, 4A-zeolite was modified with five different loadings of 5, 10, 15, 20, and 25 wt% tetraethylenepentamine (TEPA) and DEA (diethanolamine). In the preparation of 4A-25%TEPA, a solution comprising 0.33 g of TEPA and 100 mL of methanol was mixed and subjected to stirring for 20 min at 60 °C. One gram of prepared 4A-zeolite was added to the mixed solution. The solution was continuously stirred for 4 h at 500 rpm in a 100 mL beaker. The resulting mixture is dried at 100 °C for 12 h and placed in an oven to obtain a soft white powder. We repeated the process for DEA loadings of 5, 10, 15, 20, and 25 wt%. The schematic of this process is shown in Fig. 2. Prepared samples were denoted 4A-5%TEPA, 4A-10%TEPA, 4A-15%TEPA, 4A-20%TEPA, and 4A-25%TEPA. These steps are repeated for DEA.

### Characterization

The N_2_ adsorption/desorption method is a scientific method used to determine the surface area of solid materials. This test is used for measuring the adsorption of gas molecules onto the zeolite's surface at varying pressures, which is then used to calculate the specific surface area using the BET equation^60^. This test was achieved by the ASAP 2020 model at 77 K. The FTIR (Fourier Transform Infrared Spectroscopy) test is a popular analytical technique used in various fields to analyze the chemical composition of a zeolite. The test involves passing infrared radiation through a sample and measuring its absorption or transmission at different wavelengths to determine the chemical bonds present in the sample. The infrared spectrum generated from the analysis presents a distinctive identifying characteristic of the sample's composition, thus enabling the identification of unfamiliar compounds, assessment of purity, and tracking of chemical reactions. X-ray diffraction (XRD) analysis is a technique that is used for the investigation of crystal structure in materials across a broad spectrum is facilitated. This test determines the composition, purity, crystallinity, and phase identification of kaolin, zeolite, and modified zeolite^60,61^. The test device for this analysis worked at 40 mA and 40 kV. Scanning Electron Microscopy (SEM) is an electron microscopy technique that enables the acquisition of high-resolution images of the surface of a zeolite. The resulting image provides detailed information about the zeolite's morphology, topography, and composition with sub-nanometer resolution.

### Gas adsorption setup

**Figure 3:**
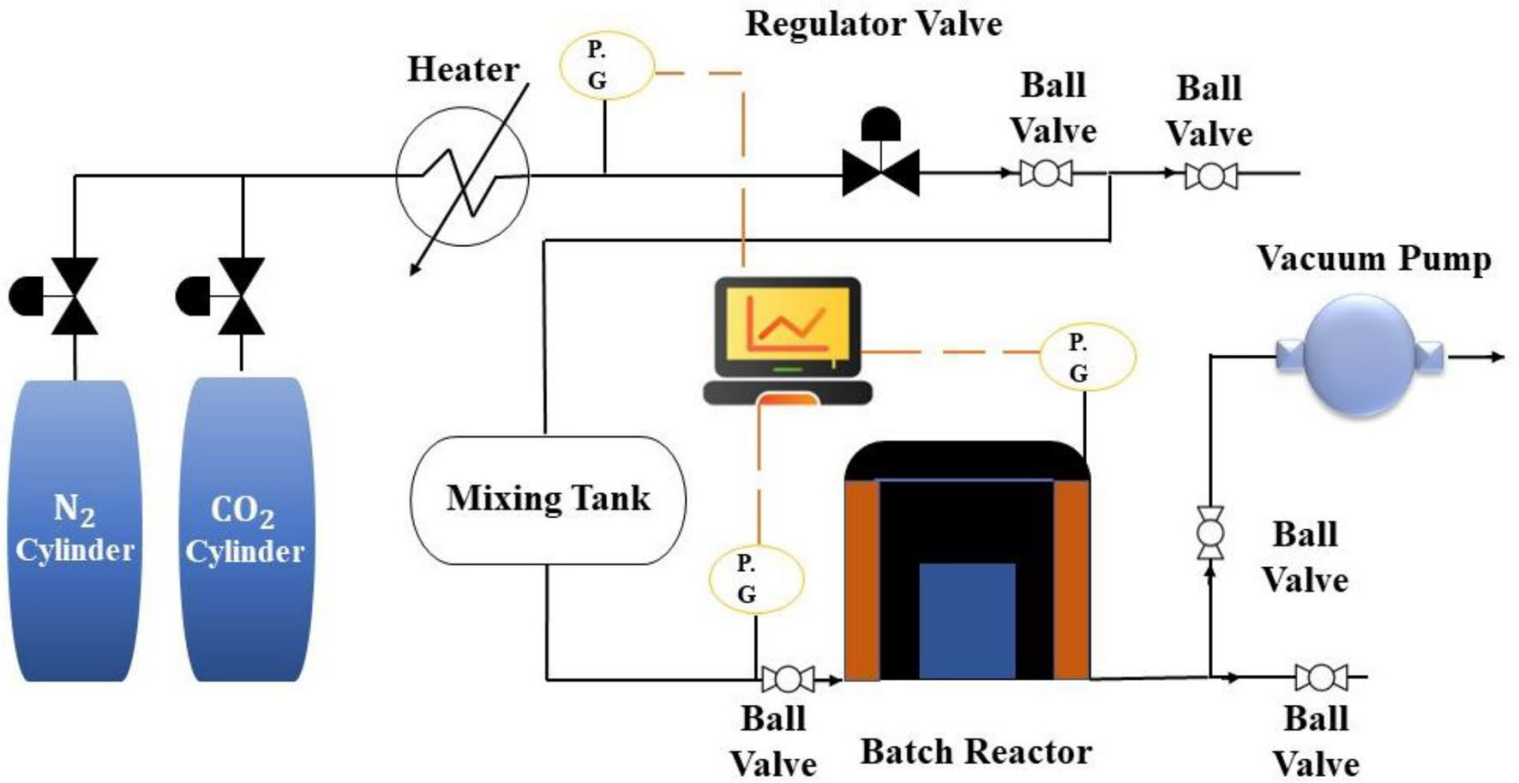
Adsorption pilot to measure CO_2_ capture.

This study evaluated the quantity of CO_2_ adsorbed on zeolites through a CO_2_ adsorption test pilot, as depicted in Fig. 3. Initially, a 0.5 g sample was loaded into the reactor, and a vacuum was produced using a vacuum pump. Subsequently, high-purity N_2_ was introduced into the chamber for 30 min before introducing CO_2_ gas, which flowed over the adsorbent for 3600 s. The experiments were performed at various pressures and temperatures. During the investigation, the temperature of the CO_2_ gas was regulated using an electric heater, and changes in temperature and pressure were continuously recorded by a computer. Upon achieving equilibrium, which took approximately one hour, the device recorded the internal pressure (P_f_). Subsequently, the adsorption parameters were determined using the recorded data^62^. The experiments were repeated three times, and the data average was reported to minimize experimental error.

The CO_2_ adsorption percentage and adsorption capacity of adsorbents are calculated using Eqs. (1) and (2), respectively.

1 $$\% \, \mathrm{ Adsorption}=\frac{{\mathrm{P}}_{\mathrm{i}}-{\mathrm{P}}_{\mathrm{f}}}{{\mathrm{P}}_{\mathrm{i}}}\times 100$$

2 $$\mathrm{q}=\frac{{\mathrm{m}}_{\mathrm{i}}-{\mathrm{m}}_{\mathrm{f}}}{{\mathrm{m}}_{\mathrm{i}}} =\frac{{(\mathrm{P}}_{\mathrm{i}}-{\mathrm{P}}_{\mathrm{f}}){\mathrm{VM}}_{\mathrm{CO}2}}{\mathrm{RTmZ}}$$

The equation involves *P*_*i*_ as the initial pressure, *P*_*f*_ as the equilibrium pressure, *V* is the reactor volume, ${M}_{CO2}$ is the molecular weight of CO_2_, R is a gas constant, m is the mass adsorbent, T is the temperature, and Z is the compressibility coefficient. The compressibility factor is obtained from virial equations [Eqs. (3)–(6)]. Table 3 displays the specific properties and calibration details of the developed unit.

**Table 3:** Specific properties and calibration details of the developed unit.

Parameters	Values	Unit
Operating temperature	298–338	K
Operating pressure	1–9	bar
Adsorbents	Zeolite 4A, Zeolite 4A + TEPA, Zeolite 4A + DEA	–
Amine concentration	5, 10, 15, 20, 25	wt%
Mass of adsorbent	0.5	g
Volume of batch reactor	4.8	mL
R	83.14472	L mbar/mol k
Gas	CO_2_	–
Gas volume	241.421	mL

3 $$\mathrm{Z}=1+\frac{\mathrm{BP}}{\mathrm{RT}}$$

4 $$\frac{{\mathrm{BP}}_{\mathrm{c}}}{{\mathrm{RT}}_{\mathrm{c}}}={\mathrm{F}}^{(0)}\left({\mathrm{T}}_{\mathrm{R}}\right)+\upomega {\mathrm{F}}^{(1)}\left({\mathrm{T}}_{\mathrm{R}}\right)$$

5 $${\mathrm{F}}^{(0)}\left({\mathrm{T}}_{\mathrm{R}}\right)=0.1445-\frac{0.330}{{\mathrm{T}}_{\mathrm{R}}}-\frac{0.1385}{{\mathrm{T}}_{\mathrm{R}}^{2}}-\frac{0.0121}{{\mathrm{T}}_{\mathrm{R}}^{3}}-\frac{0.000607}{{\mathrm{T}}_{\mathrm{R}}^{8}}$$

6 $${\mathrm{F}}^{(1)}\left({\mathrm{T}}_{\mathrm{R}}\right)=0.0637+\frac{0.331}{{\mathrm{T}}_{\mathrm{R}}^{2}}-\frac{0.423}{{\mathrm{T}}_{\mathrm{R}}^{3}}-\frac{0.008}{{\mathrm{T}}_{\mathrm{R}}^{8}}$$

### Response surface methodology (RSM)

RSM is the statistical technique to model and optimize complex relationships between multiple input variables and output responses^56^. The investigation conducted in this research involved an examination of the effects of various factors, such as temperature, pressure, and loading percentage of two different amines on 4A-zeolite, to enhance the performance of CO_2_ capacity. RSM was implemented to optimize these factors, utilizing a central composite design (CCD) based on a four-factor approach, which included temperature (A), pressure (B), amine wt% (C), and amine type (D), as detailed in Table 4.

**Table 4:** The process factors in the RSM modeling for 4A-zeolite with TEPA and DEA.

Factor	Symbol	Name	Units	Type	Sub type	Minimum	Maximum	Mean	Std. dev
Temperature	A	T	C	Numeric	Continuous	25.00	65.00	45.00	7.92
Pressure	B	p	bar	Numeric	Continuous	1.0000	9.00	5.00	1.58
Wt% amine	C	% loading	%	Numeric	Continuous	5.00	25.00	15.00	3.96
Type of amine	D	D		Categoric	Nominal	TEPA	DEA	Levels:	2.00

Total of 52 tests (Table S1 in the supplementary) were conducted under varying operating conditions, and the resulting experimental data was used to establish the relationship between the X variable and the Y response through a design model [Eq. (7)]

7 $$y={a}_{0}+{\sum }_{i=1}^{n}{a}_{i}{x}_{i}{\sum }_{i=1}^{n}{a}_{ii}{x}_{i}^{2}+{\sum }_{i}^{n}{\sum }_{j}^{n}{a}_{ij}{x}_{i}{x}_{j}+\varepsilon$$

In this formula [Eq. (7)] y is the dependent variable, a_0_ is the intercept, a_i_ is the regression coefficient for the n predictor variable x_i_, and a_ii_ is the coefficient for the squared term of each predictor, a_ij_ is the coefficient for the interaction terms, and ε represents the residual error term. The formula suggests that the dependent variable y is a function of the importance of the predictor variables, the regression coefficients, and the error term. In linear regression, the aim is to determine the values of the regression coefficients that minimize the sum of the squared differences between the predicted and actual values of y. RSM has been used to predict experimental data by fitting a mathematical model to the observed data, which can then be used to make predictions for new combinations of input variables. By employing this approach, we can ascertain the optimal conditions necessary to achieve a desired response and assess the responsiveness of the response to variations in the input variables.

## Results and discussion

### Characterization results

**Figure 4:**
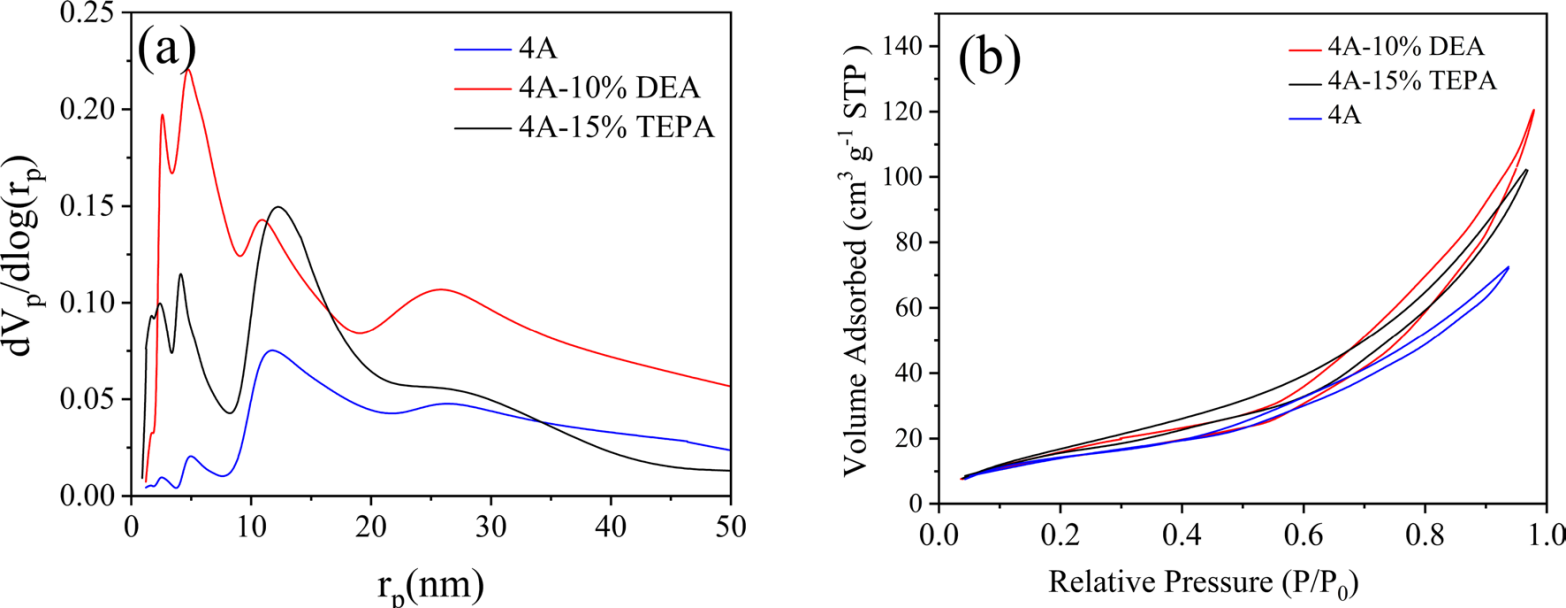
(**a**) The pore-size distribution by BJH method for adsorbent samples, (**b**) N_2_ adsorption/desorption at 77K.

N_2_ adsorption–desorption results of 4A-zeolite and modified zeolites indicate in Table 5 and Fig. 4b. The pore size distribution of zeolite 4A, 4A-TEPA, and 4A-DEA was analyzed using the Barrett–Joyner–Halenda equation (BJH)^63^, and the results are present in Fig. 4a. The BJH pore diameters for 4A, 4A-TEPA, and 4A-DEA were determined to be 11.1 nm, 10.1 nm, and 6 nm, respectively. Table 5 provides information on the micropore volume, BET surface area, and micropore surface area of the zeolite 4A and modified zeolites.

**Table 5:** BET Characterization of zeolite 4A and its modified structures.

Adsorbent	S_BET_ (m^2^/g)	Pore size (nm)	Total pore volume (cm^3^/g)
4A	70.3	10.123	0.2167
4A-DEA	52.563	6.342	0.1802
4A-TEPA	46.02	9.107	0.1390

**Figure 5:**
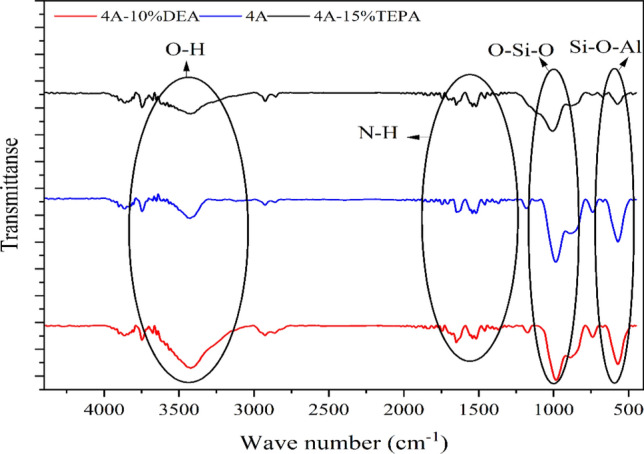
FT-IR of zeolite 4A, Zeolite 4A-TEPA, and Zeolite 4A-DEA.

The FT-IR spectrum of Fig. 5 shows the comparison of three samples: 4A-zeolite, 4A-15%TEPA, and 4A-10%DEA. In the spectrum of the commercial 4A-zeolite there are several prominent peaks and troughs that can be identified and analyzed. The peak at around 3429 cm^−1^ corresponds to the O–H stretching vibration of adsorbed water molecules, indicating the presence of water in the zeolite. In the spectrum of the modified zeolite with DEA and TEPA, some several additional peaks and troughs can be identified and analyzed. The peak at around 3417 cm^−1^ corresponds to the O–H stretching vibration of adsorbed water molecules, similar to the commercial zeolite 4A. The peak at around 1544 cm^−1^ corresponds to the N–H bending vibration of the amine groups, further confirming the presence of the amines^64^. Comparing the spectra of the modified zeolites with the unmodified 4A-zeolite reveals changes in peak intensity and position. For the FT-IR structure of 4A-zeolite, the vibration bands at 1001 cm^−1^ and 570 cm^−1^ could be assigned to the stretching vibration of Si–O or Al–O units and the vibration of Si–O–Al units in the 4A-zeolite structure, respectively^63^.

**Figure 6:**
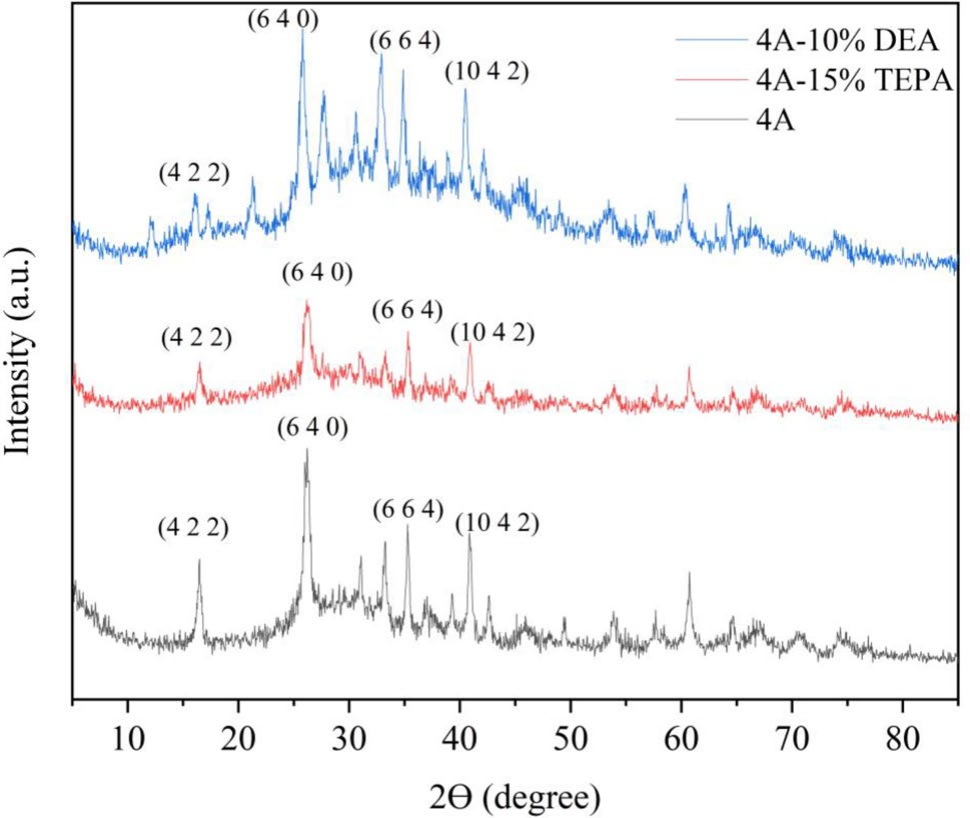
XRD profiles of Zeolite 4A, Zeolite 4A-TEPA, and Zeolite 4A-DEA.

Figure 6 shows the XRD pattern of samples. For zeolite 4A and amine-modified Zeolites, the positions of characteristic peaks are consistent, the same as based 4A-zeolite. The most intense peaks occur at 17.2°, 26.17°, 35.1°, 40.2°, and 60° 2θ, which can be indexed to an amorphous structure consistent with zeolite 4A because it was synthesized from clay materials^65^. All zeolites exhibit additional peaks when compared to the untreated 4A-zeolite. The samples used for 4A, 4A-TEPA, and 4A-DEA were free of impurities, as confirmed by the similarities observed in their XRD patterns.

**Figure 7:**
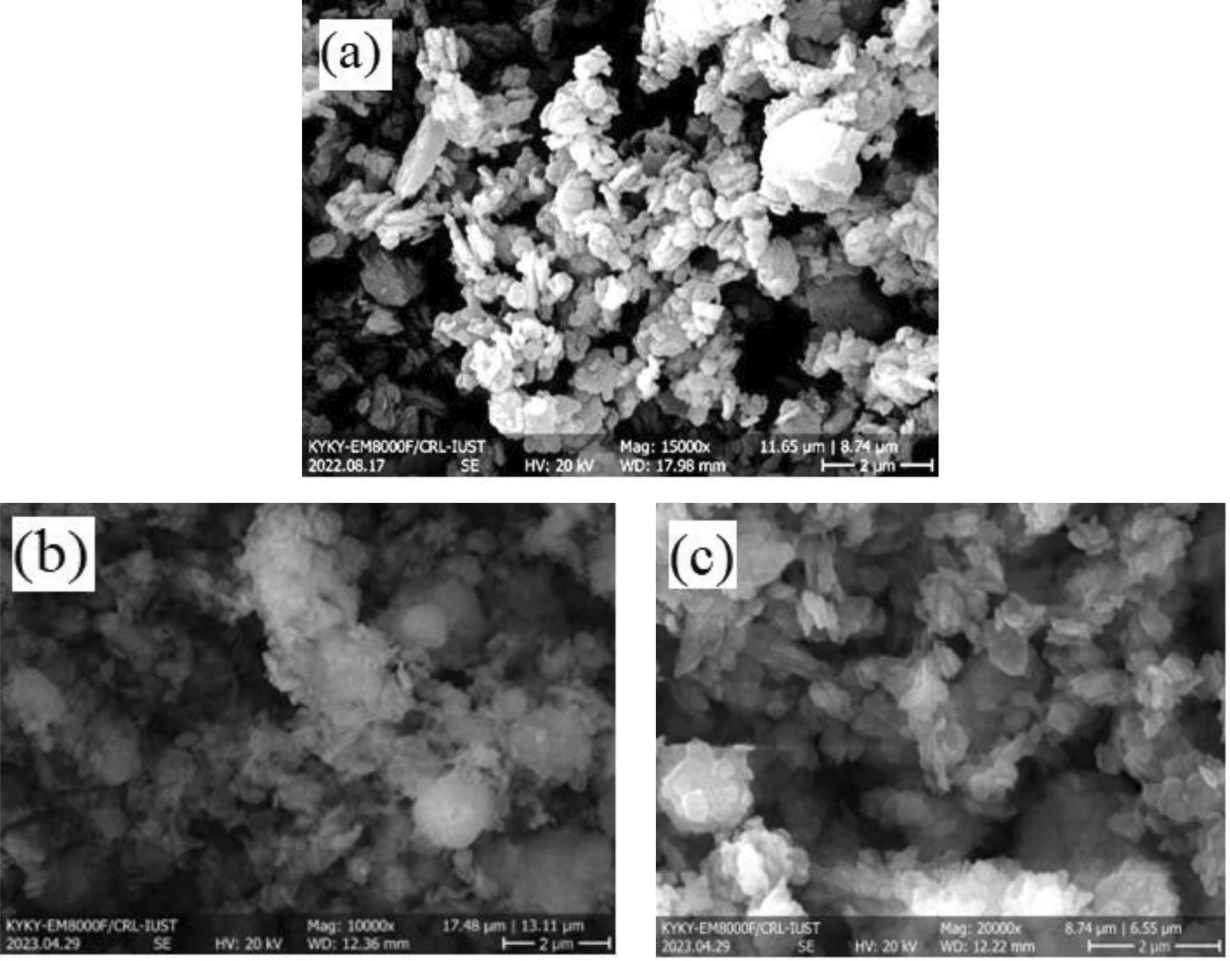
SEM images of (**a**) Zeolite 4A, (**b**) Zeolite 4A-10%DEA, and (**c**) Zeolite 4A-15%TEPA.

In Fig. 7a, the bright and dark areas represent variations in the topography and composition of the zeolite 4A surface. The bright regions correspond to high points on the surface, while the dark areas correspond to low points or pores within the zeolite structure. In Fig. 7b and c, the presence of pores within the zeolite structure emerges as a critical characteristic of this material, as it offers sites for the adsorption of CO_2_. The effect of amine in 4A-zeolite determines in Table 5.

### RSM results

This research study utilized response surface methodology (RSM) based on central composite design (CCD) to explore and optimize CO_2_ adsorption by two modified zeolite 4A adsorbents with TEPA and DEA. The investigation employed a factorial design incorporating four factors temperature, pressure, the percentage of amine used for surface modification, and the type of amine, resulting in a total of 52 tests. The study findings include the values of the independent factors and the CO_2_ adsorption capacity. The amount of change shown by the response variables in the assimilation process indicates the magnitude and direction of their influence, as determined by the corresponding signs. The values about the TEPA and DEA factors were obtained using Eqs. (8, 9), respectively, providing a practical means to explain the resultant predictive relationship.

8 $${q}_{C{O}_{2}}(\mathrm{TEPA})=139.42197-5.89412\times T\text{+ 43.07921}\times P\text{+ 0.478457}\times C-\text{0.487504}\times T\times P -0.091647\times T\times C+0.824998\times P\times C+0.108529\times \left({T}^{2}\right)+1.05560\times \left({P}^{2}\right)+0.042884\times ({C}^{2})$$

9 $${q}_{C{O}_{2}}(\mathrm{DEA})=+274.43678-9.03449\times T\text{+ 48.61391}\times P-1.41403\times C-\text{0.487504}\times T\times P -0.091647\times T\times C+0.824998\times P\times C+0.108529\times \left({T}^{2}\right)+1.05560\times \left({P}^{2}\right)+0.042884\times ({C}^{2})$$

It is imperative to assess the significance of the model, its independent parameters, and any interactions and second-order terms that may exist within it for a desirable response.

The ANOVA analysis Table 6. ANOVA model of adsorption capacity Presents statistical data concerning the parameters of temperature, pressure, amine loading, CO_2_ adsorption capacity, and efficiency. Statistical analysis conducted on the model yielded a Model F-value of 34.19, which suggests that the model is significant. The likelihood of an F-value of this magnitude occurring due to random error is only 0.01%, indicating a high degree of confidence in the model's validity. In line with established conventions, model terms with p-values below 0.0500 consider statistically significant^36^, while those with p-values greater than 0.1000 deem insignificant. Accordingly, in the present analysis, the terms A, B, AD, and A^2^ consider significant contributors to the model.

**Table 6:** ANOVA model of adsorption capacity.

Source	Sum of squares	df	Mean square	F-value	p-value
Model	3.096E+05	13	23,816.40	34.19	< 0.0001
A-T	7285.47	1	7285.47	10.46	0.0025
B-p	2.808E+05	1	2.808E+05	403.20	< 0.0001
C-% loading	537.42	1	537.42	0.7716	0.3852
D–D	639.86	1	639.86	0.9187	0.3439
AB	1521.03	1	1521.03	2.18	0.1477
AC	335.96	1	335.96	0.4824	0.4916
AD	7889.54	1	7889.54	11.33	0.0018
BC	1088.99	1	1088.99	1.56	0.2188
BD	980.25	1	980.25	1.41	0.2429
CD	716.30	1	716.30	1.03	0.3169
A^2^	7046.70	1	7046.70	10.12	0.0029
B^2^	1066.62	1	1066.62	1.53	0.2235
C^2^	68.76	1	68.76	0.0987	0.7551
Residual	26,467.24	38	696.51		
Lack of fit	26,467.24	16	1654.20		
Pure error	0.0000	22	0.0000		
Cor TOTAL	3.361E+05	51			

**Figure 8:**
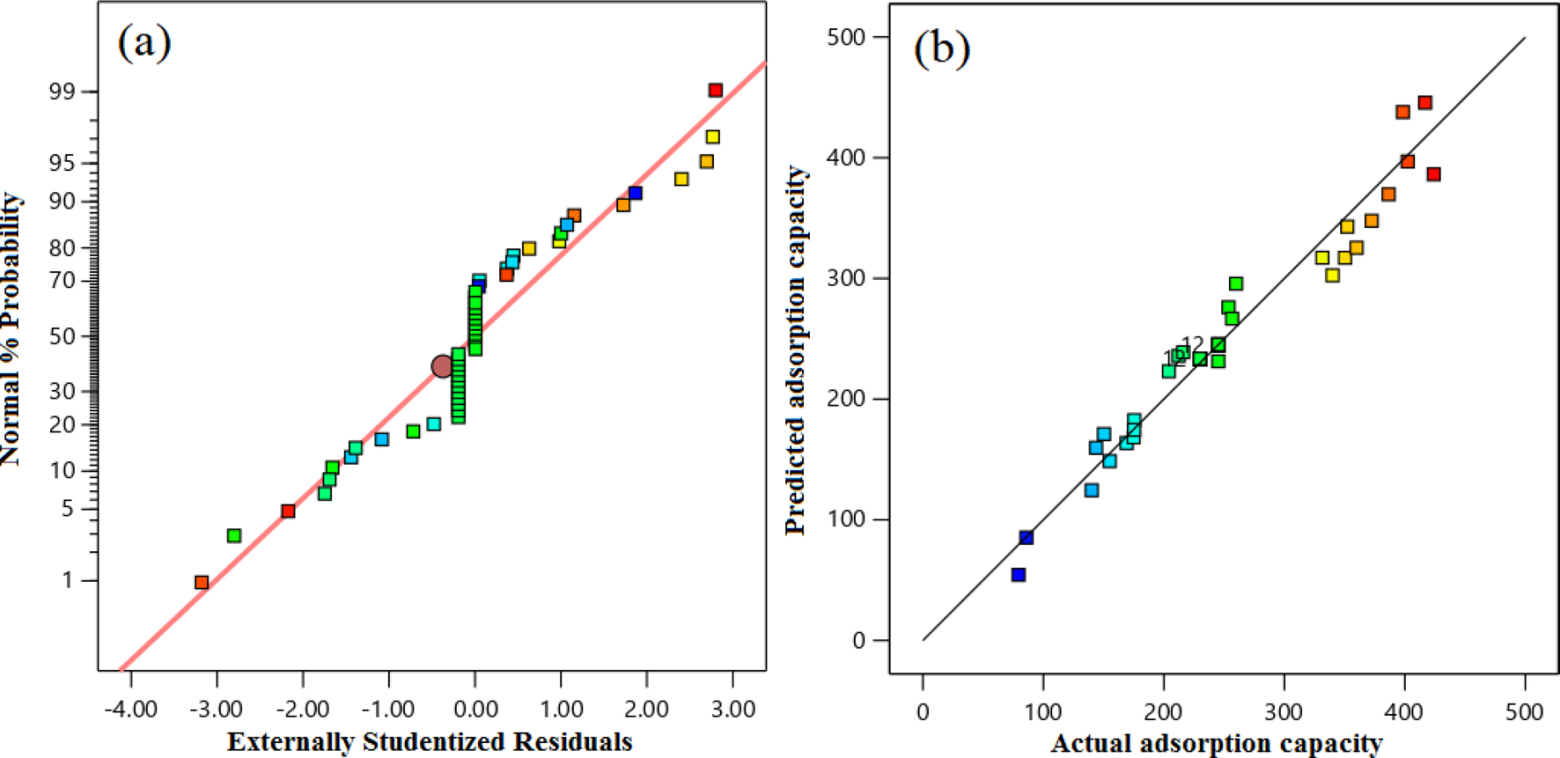
The CCD forecasted the CO_2_ capture capacity estimated value in comparison to (**a**) the normal probability and (**b**) the actual capture capacity.

In contrast, those with p-values greater than 0.1000 are deemed to have no significant impact. The model terms of temperature and pressure have F-values of 10.46 and 403.20, respectively. In other words, the high F-values provide evidence that the model is meaningful, as the model terms have a substantial influence on the response variable. The correlation coefficient (0.9212) obtained for the CO_2_ uptake capacity indicates a satisfactory agreement between the correlation coefficients and experimental data. The difference between the predicted R^2^ of 0.7300 and the adjusted R^2^ of 0.8943 is within 0.2, indicating a good match. Additionally, Adeq Precision, which gauges the signal-to-noise ratio, should exceed 4, But in this case, it measures 28.981, indicating an adequate signal. Hence, this model can be applied to explore the design space. Ratio more significant than 4 is desirable; a ratio of 28.981 indicates an adequate signal. This model can be used to navigate the design space. The performance of a proposed model for CO_2_ adsorption evaluates using residual plots and a comparison of actual and predicted values. Figure 8a displays the residual field, which depicts the deviation between actual and predicted values of the model's responses. The degree of appropriateness and normality displayed by the distribution of data points surrounding the linear regression line in this plot indicates whether the errors distribute adequately. Figure 8b shows a story of the actual and predicted values, revealing a good agreement between the two. This result suggests that the proposed model can accurately predict the amount of CO_2_ adsorption under various operating conditions.

**Figure 9:**
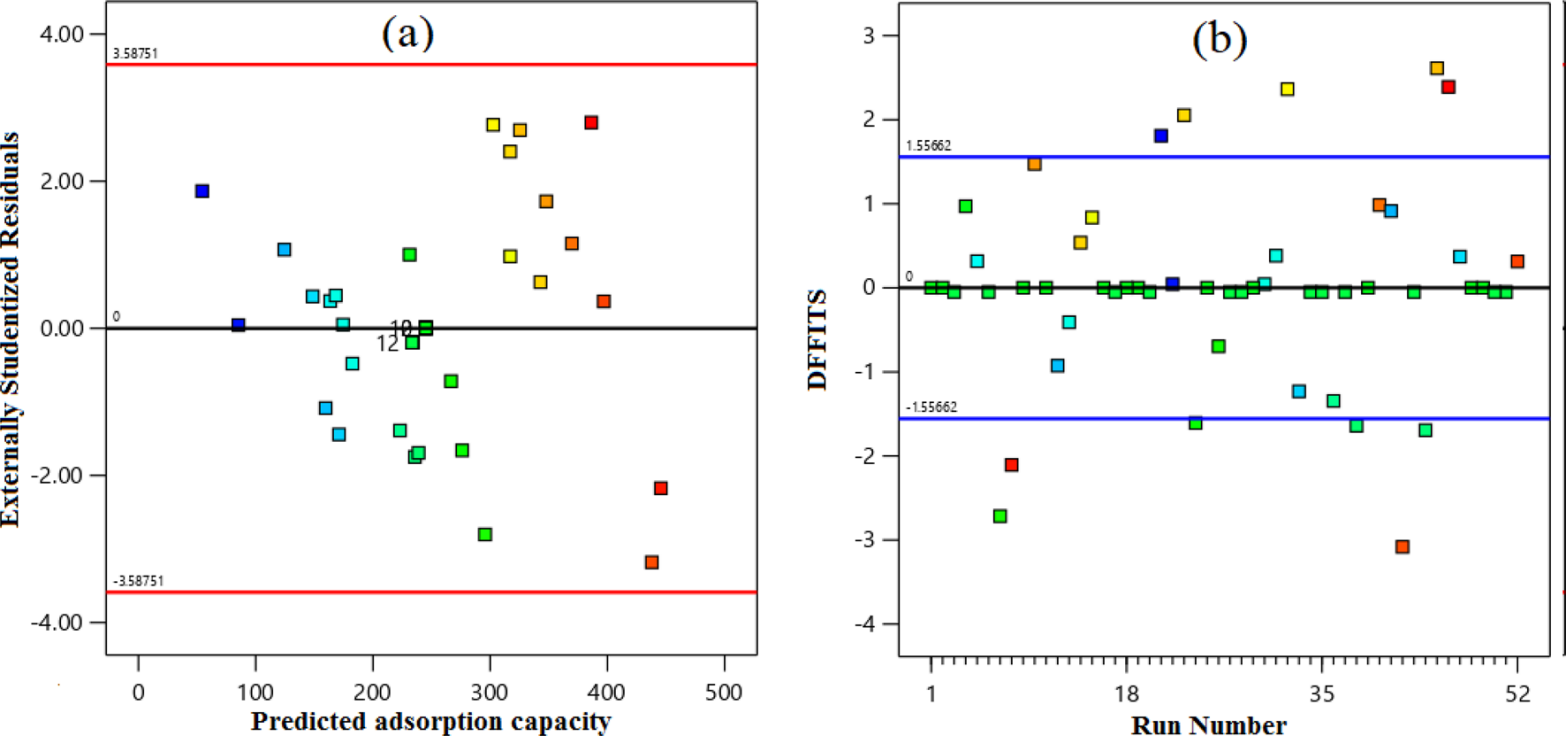
The predicted CO_2_ uptake capacity in relation: (**a**) Externally studentized residual, and (**b**) Run number.

In order to further assess the reliability of the final model, a residual plot was generated by plotting the predicted response values against the residuals, as shown in Fig. 9a. The resulting plot displayed a scattered distribution of points along the x-axis, ranging from + 3.58751 to − 3.58751, with no discernible trends. This observation suggests that the models were adequate and reliable, with a consistent variance observed across the range of responses. Moreover, this approach served as an additional tool for evaluating the suitability of the final model. We utilized the method of tracing predicted responses toward the model residuals, as illustrated in Fig. 9a and b, to examine whether there were any notable levels of constant variance.

**Figure 10:**
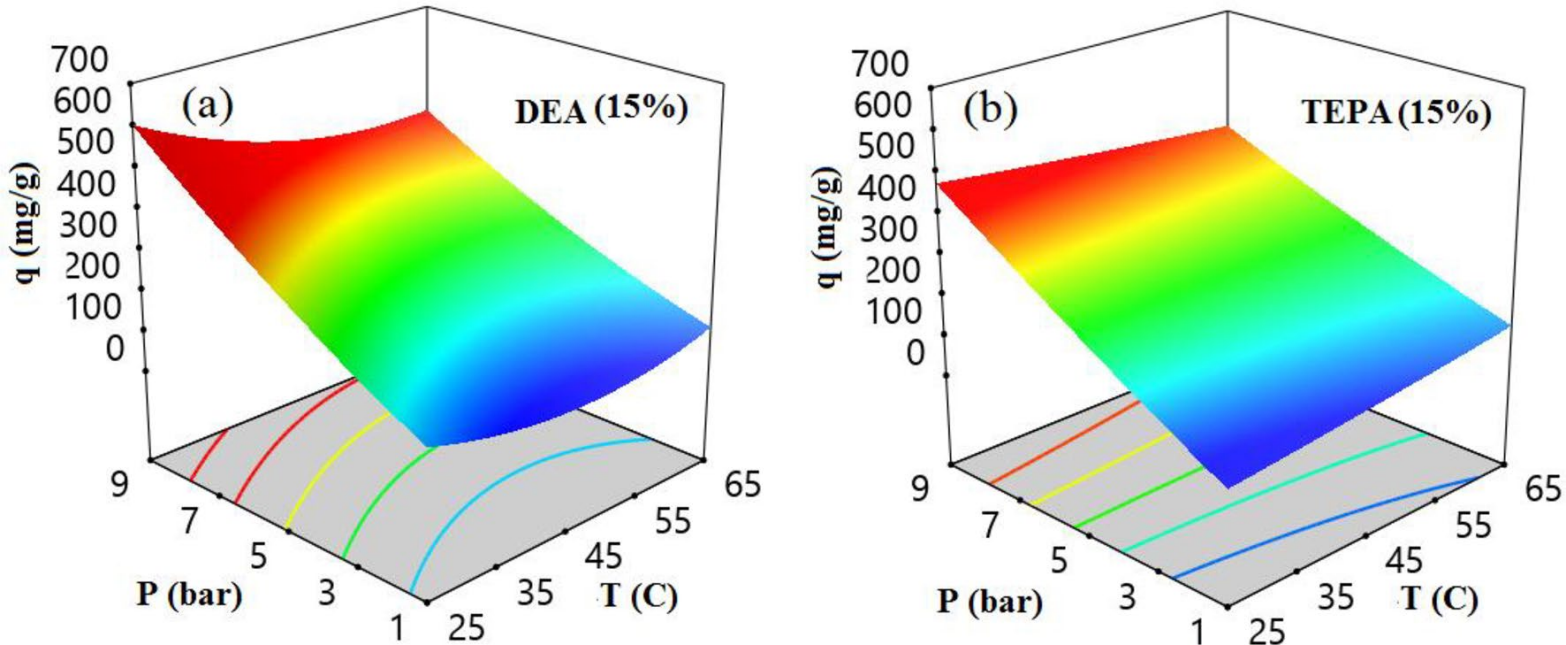
Interactions between P*T and their impact on CO_2_ adsorption capacity (**a**) Zeolite 4A-DEA and (**b**) Zeolite 4A-TEPA.

Researchers in the RSM often use three-dimensional response surfaces to study and determine the best conditions. These surfaces can analyze the connections between factor variables and responses. Researchers can examine the impact of variables on a system in great detail by focusing on the response functions of two parameters while keeping all other parameters constant. Figure 10 presents a three-dimensional diagram, obtained using response surface methodology, for two zeolite modifications with DEA (Fig. 10a) and TEPA (Fig. 10b), depicting the impact of the interaction between pressure and temperature variables on the CO_2_ adsorption capacity of modified-zeolites. We evaluated both zeolite modifications at different temperatures and pressures, ranging from 25 to 65 °C and 1 to 9 bar, respectively. Afterward, we analyzed the obtained data using Design Expert software. The analysis revealed that an increase in pressure caused increasing in CO_2_ adsorption for both modifications, while higher temperatures resulted in a reduction in adsorption capacity. We also examined an amine concentration of 15% for both adsorbents.

#### Deviation plots

**Figure 11:**
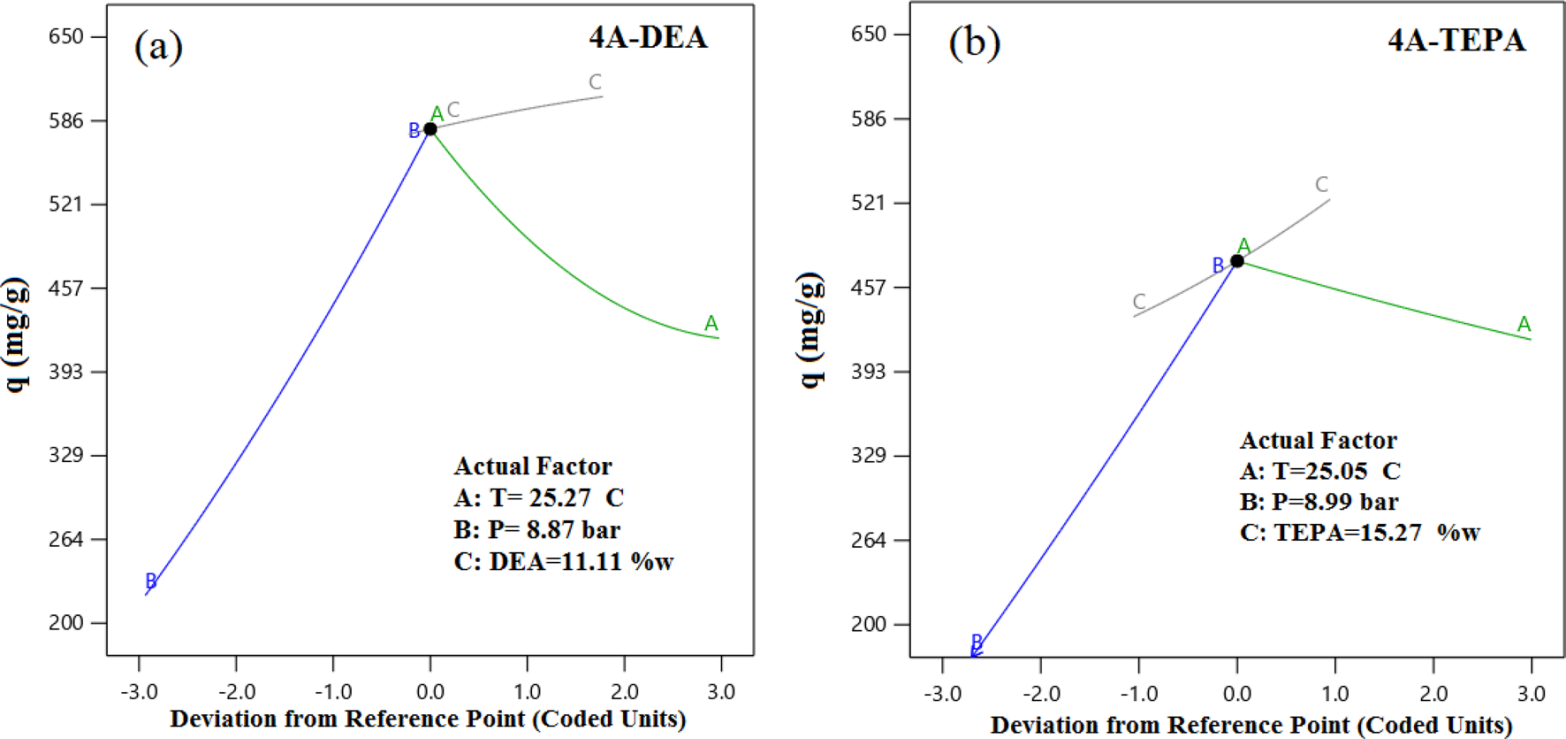
Deviation curves for responses of two kinds of modified zeolite with coded factors for (**a**) 4A-DEA, and (**b**) 4A-TEPA.

The deviation plot illustrates the comprehensive impact of all process parameters on the response function, with the central point (0) serving as the midpoint of the operating range. This outcome offers valuable insights into the overall behavior of the studied system. Figure 11 presents a perturbation plot highlighting the effect of all four operating parameters, namely temperature, pressure, and wt% amine, at the reference points. The results reveal that an increase in temperature (A) and wt% amine (C) leads to a decrease in the CO_2_ capacity of DEA, whereas an increase in pressure (B) enhances CO_2_ capture. Furthermore, Fig. 11a and b demonstrates that TEPA and DEA exhibit similar behavior under the experimental conditions tested.

#### Optimum adsorption operating parameters

This study aimed to identify the optimal combination of independent variables, namely pressure, temperature, and wt% of amine, to achieve maximum adsorption performance. The Response Surface Methodology (RSM) optimization technique is proposed by conducting a series of tests. Input parameters were given ranged values to achieve the maximum response of CO_2_ adsorption capacity. Tables 7 and 8 present the limiting conditions for zeolites modified with DEA and TEPA amines, respectively.

**Table 7:** Optimization of condition for 4A-DEA.

Name	Goal	Lower limit	Upper limit	Lower weight	Upper weight	Importance
A: T (°C)	Is in range	25	65	1	1	3
B: p (bar)	Is in range	1	9	1	1	3
C: % loading	Is in range	5	25	1	1	5
D: amine type	Is equal to DEA	TEPA	DEA	1	1	3
q (mg/g)	Maximize	79.42	424.029	1	1	3
% adsorption	Maximize	5.2	10.32	1	1	5

**Table 8:** Optimization of condition for 4A-TEPA.

Name	Goal	Lower limit	Upper limit	Lower weight	Upper weight	Importance
A: T (°C)	Is in range	25	65	1	1	3
B: p (bar)	Is in range	1	9	1	1	3
C: % loading	Is in range	5	25	1	1	5
D: AMINE TYPE	Is equal to TEPA	TEPA	DEA	1	1	3
q (mg/g)	Maximize	79.42	424.029	1	1	5
% adsorption	Maximize	5.2	10.32	1	1	5

### Optimization of condition for 4A-DEA and 4A-TEPA

Determining the optimal operating and structural conditions for modified zeolites to achieve the maximum CO_2_ adsorption capacity is the main objective of this study. Optimizing variable components is one of the methods to enhance CO_2_ adsorption efficiency in modified zeolites. Table 9 presents the optimal values and specified ranges for the CO_2_ adsorption process when TEPA and DEA utilize to modify 4A-zeolite.

**Table 9:** The optimal values of uptake optimization results by zeolite-modified adsorbents.

Adsorbent	T	P	% loading	D	q	% adsorption
4A-TEPA	25.050	8.991	15.275	TEPA	477.342	10.397
4A-DEA	25.270	8.870	11.112	DEA	579.468	10.325

After obtaining the optimal conditions, we plan to perform isotherm, kinetic, and thermodynamic modeling on the modified zeolites.

### Influence of amine loading quantity on CO_2_ adsorption capacity

**Figure 12:**
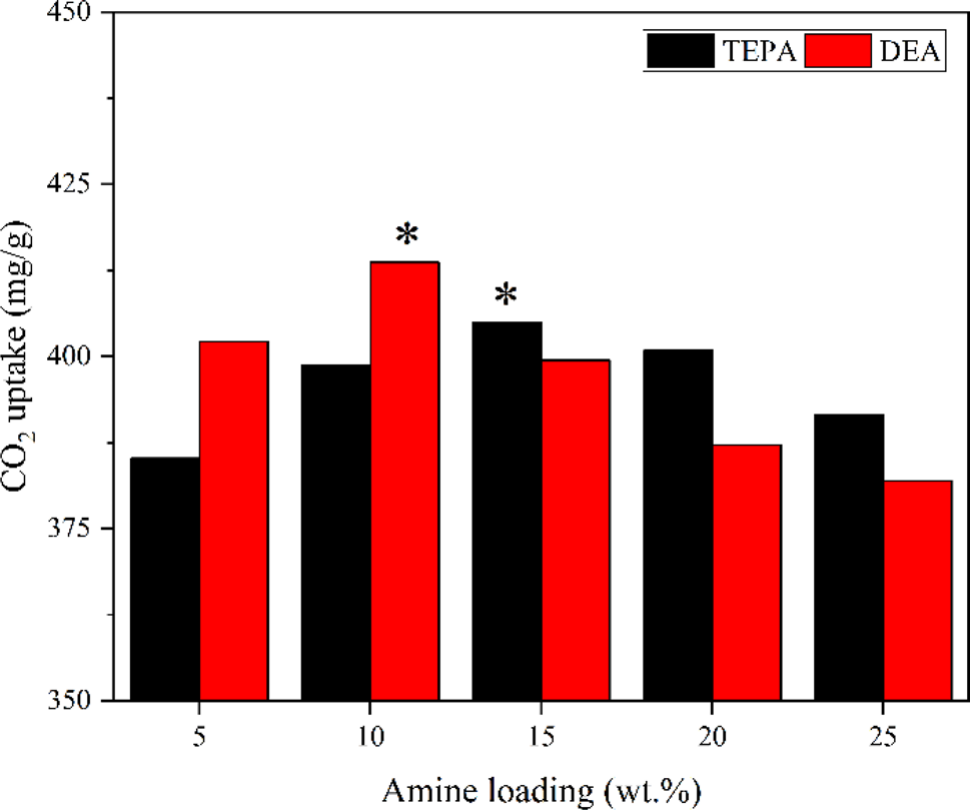
CO_2_ adsorption capacity of the adsorbents at different DEA and TEPA loadings.

Amines are commonly used as functional groups on adsorbent materials due to their ability to interact with CO_2_ molecules through chemisorption, resulting in enhanced CO_2_ capture performance. By incorporating amine functional groups, more sites for CO_2_ adsorption are introduced that improve the adsorption capacity through increased surface interactions. Amine loading plays a direct role in CO_2_ adsorption efficacy as amines are the primary active sites for CO_2_ adsorption in solid adsorbents that are amine-based and functionalized. Very high loading of amine functional groups can lead to steric hindrance, limiting access to the amine sites and reducing CO_2_ adsorption efficiency^66^. Therefore, careful optimization of TEPA or DEA loading is essential to balance the benefits of increased loading without negatively affecting adsorption performance. The CO_2_ adsorption capacity of the adsorbents at different DEA and TEPA loadings is shown in Fig. 12. According to Fig. 12, the ideal DEA and TEPA loading for 4A-zeolite were 10 and 15 wt%, respectively. Due to the low DEA load and the decreased tendency of pore-clogging in 4A-10%DEA, the adsorption capacity is the highest^67^. The broadest pore size distribution, which enhances TEPA dispersion and CO_2_ molecule diffusion, is 4A-15%TEPA (Fig. 4). The best CO_2_ adsorption capacity is found in 4A-10%DEA, which has more micropores (Table 5) hence lowers the resistance to mass transfer and boosts the capacity for CO_2_ adsorption^68^.

### Isotherm modeling

Utilizing isotherm modeling is crucial in investigating CO_2_ adsorption in amine-modified zeolites, as it provides a quantitative description of the adsorption behaviours of CO_2_ on the modified zeolite surface. The Langmuir Eq. (10), Freundlich Eq. (11), and Dubinin-Radushkevich (D–R) Eq. (12) models were used in this study to describe the behaviour, as they are among the various isotherm models available.

10 $${q}_{e}=\frac{{q}_{m}{K}_{L}{P}_{e}}{1+{K}_{L}{P}_{e}}$$

11 $${q}_{e}={K}_{F}{P}_{e}^\frac{1}{n}$$

12 $${q}_{e}={q}_{m}{e}^{-\lambda {\omega }^{2}}$$

The parameters q_e_ and q_m_ represent the equilibrium and maximum adsorption capacities of CO_2_, respectively, and measure in units of mg/g. The Langmuir isotherm model is characterized by the parameter K_L_, which represents the Langmuir equilibrium constant (bar^−1^). The Freundlich isotherm model is characterized by the parameters k_F_ (mg g^−1^ bar^−1/n^), P_e_ (bar), and n (Freundlich isotherm constant). The D–R isotherm model characterizes by two parameters, namely the constant of the model (λ) in mol^2^/J^2^, and the Polanyi potential (ω) in KJ/mol units.

**Figure 13:**
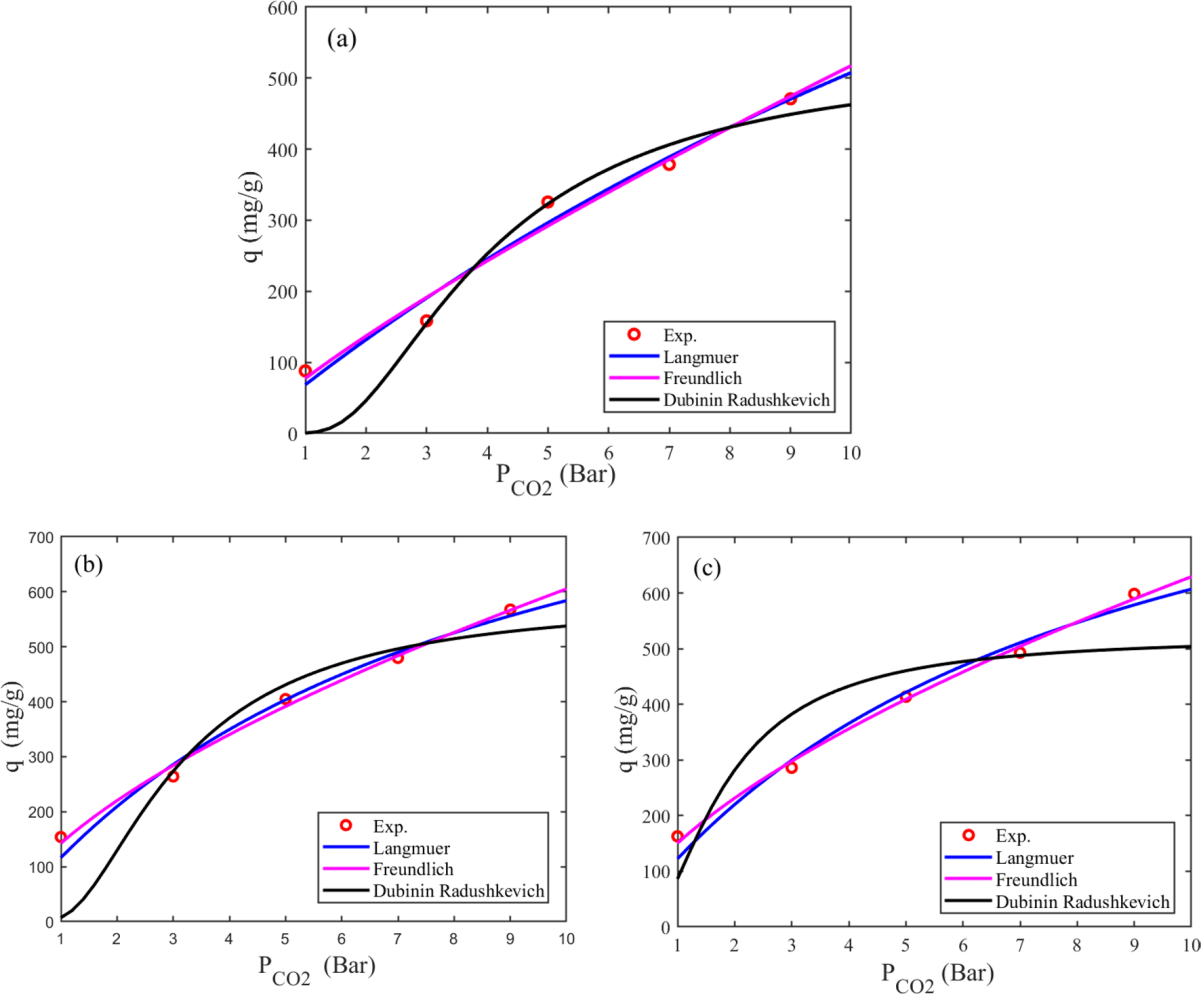
Comparison of isothermal models and experimental values of CO_2_ adsorption at the temperature of 298 K and pressure of 5 bar by (**a**) 4A, (**b**) 4A-15%TEPA, and (**c**) 4A-10%DEA.

CO_2_ adsorption isotherms using these models plot at 298 K and pressures ranging from 1 to 9 bar, as depicted in Fig. 13a–c. The results indicated that an increase in uptake pressure led to a rise in the rate of CO_2_ adsorption. Table 10 presents the experimental findings and the R^2^ correlation coefficients for all coefficients of isotherm parameter models. Based on the nonlinear regression technique and the R^2^ values, the theoretical isotherms rank in order of effectiveness for explaining and predicting adsorption for the behavior of modified zeolite as Freundlich > Langmuir > D–R. The Freundlich isotherm model's ability to fit well with the adsorption data indicates that the modified zeolite surface is not uniform and has a wide range of adsorption energies. This behavior explains the heterogeneous surface with broad adsorption energy distribution through the Freundlich constant and exponent parameters. A high Freundlich constant shows that the modified zeolite has a high adsorption capacity, while a low exponent means a more linear adsorption isotherm. In conclusion, the Freundlich isotherm model provides valuable information about CO_2_ adsorption on amine-modified zeolites and can help optimize their design and performance for CO_2_ capture applications.

**Table 10:** Theoretical isotherm models of CO_2_ adsorption at 5 bar and 298 K.

Model	Parameters	Adsorbent		
		4A	4A-15%TEPA	4A-10%DEA
Langmuir	q_m_	1770.081	1056.175	1084.177
	k_l_	0.040	0.124	0.127
	R^2^	0.9882	0.9923	0.9915
Freundlich	K	77.246	142.406	150.381
	n	1.211	1.591	1.609
	R^2^	0.9879	0.9967	0.9978
Dubinin–Radushkevich (D–R)	q_s_	529.481	584.404	521.389
	$\uplambda$	2.421	1.493	0.610
	$\upomega$	0.454	0.579	0.905
	R^2^	0.9826	0.9598	0.8865

### Kinetics modeling

The analysis of the adsorption rate, through kinetics, is crucial in determining the required residence time for evaluating the adsorption reaction. In the study of adsorption data, two primary categories of mathematical models are commonly utilized: adsorption reaction models and adsorption diffusion models. Although both models describe the kinetic process of adsorption, they represent different aspects of the kinetic analysis^69^. In the case of reaction models, experimental data fit with differential equations such as pseudo-first order, pseudo-second order, etc. (Table 11), which help to determine the reaction order and rate constants^46^. In contrast, adsorption diffusion models base on three consecutive steps: external diffusion or film diffusion (i.e., diffusion across the gas film surrounding the adsorbent particle), internal diffusion or intraparticle diffusion (i.e., diffusion of gas in the pores and, or along the pore walls), and mass action (i.e., adsorption and desorption between gas molecules and active sites)^69^. The variables q_t_, k_1_, k_2_, and k_A_ use represent the adsorption capacity and rate constants of the first-order, second-order, and fractional-order models. In addition to n, a, and b use employing mean the kinetic model parameters, that important in characterizing the adsorption process's kinetic behavior and understanding the underlying mechanisms.

**Table 11:** Fitting parameters of kinetic models.

Model	Equation	Parameters	Adsorbent					
			4A		TEPA		DEA	
			298 K	308 K	298 K	308 K	298 K	308 K
First order	${\mathrm{q}}_{\mathrm{t}}=(1-{\mathrm{e}}^{({\mathrm{k}}_{1}\mathrm{t})})$	${q}_{e}$	289.676	272.772	359.449	282.089	369.117	317.957
		${k}_{1}$	0.234	1.159	2.567	10.280	0.766	10.425
		R^2^	0.860	0.60123	0.50506	0.63691	0.60579	0.74588
Second order	${\mathrm{q}}_{\mathrm{t}}={\mathrm{q}}_{\mathrm{e}}-\frac{{\mathrm{q}}_{\mathrm{e}}}{{1+{\mathrm{k}}_{2}\mathrm{tq}}_{\mathrm{e}}}$	${q}_{e}$	315.464	286.161	371.680	285.553	387.439	320.98
		${k}_{2}$	0.0012533	0.005057 0.82016	0.006492	0.0325399	0.0026608	0.037613
		R^2^	0.91765		0.76572	0.77190	0.82065	0.86436
Fractional order	${\mathrm{q}}_{\mathrm{t}}={\mathrm{q}}_{\mathrm{e}}(1-{\mathrm{e}}^{{-({\mathrm{k}}_{\mathrm{A}}\mathrm{t})}^{{\mathrm{n}}_{\mathrm{A}}}})$	${q}_{e}$	282.249	230.5903	215.338	260.990	262.582	349.959
		${k}_{n}$	0.0074	0.0088	0.1725	0.0090	0.2458	0.3843
		m	0.2065	0.1239	0.1022	0.0626	0.1255	0.1261
		n	1.0525	1.0259	0.6665	1.0366	0.6187	0.8739
		R^2^	0.99337	0.98713	0.97470	0.97589	0.98680	0.97546
Intraparticle Diffusion	${\mathrm{q}}_{\mathrm{t}}={\mathrm{k}}_{\mathrm{int}}{\mathrm{t}}^\frac{1}{2}$	${k}_{c}$	54.397	55.305	70.715	56.989	64.740	63.881
		R^2^	0.99263	0.94855	0.95687	0.83887	0.96628	0.70449
Elovich	${\mathrm{q}}_{\mathrm{t}}=\frac{1}{\mathrm{b}}\mathrm{ Ln}(1+\mathrm{abt})$	$\alpha$	0.5178	29.600418	18.892	76.88	13.882	68.185
		$\beta$	44.476	28.164	30.920	14.784	38.652	12.372
		R^2^	0.97735	0.97420	0.96795	0.95672	0.97971	0.94838

**Figure 14:**
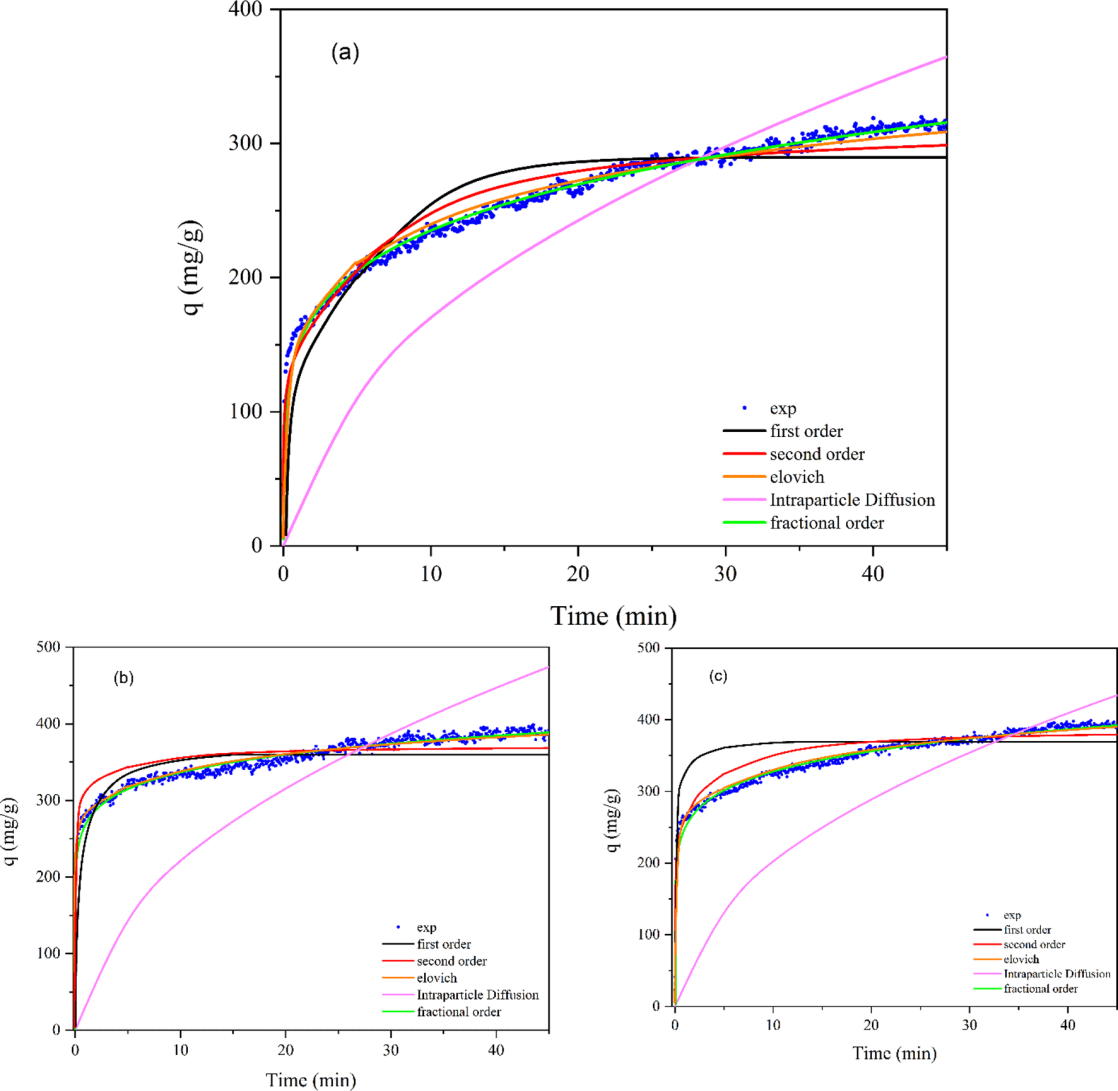
Comparison of kinetic models and experimental values of CO_2_ adsorption at the temperature of 298 K and pressure of 5 bar by (**a**) 4A, (**b**) 4A-15%TEPA, and (**c**) 4A-10%DEA.

The first-order model assumes that the rate of solute uptake changes proportionally to the difference in saturation concentration and the amount of solid uptake over time, indicating a physical adsorption process. If the R^2^ value of the latter model decreases, as shown in Table 11, it suggests that chemical adsorption plays an increasingly important role in the adsorption processes^70^. The Rate Controlling Model has been commonly used to analyze mass transfer mechanisms and has established intraparticle diffusion as the sole determining factor in regulating the process rate^71^. Based on the data presented in Table 11 and the correlation coefficient (R^2^) values of the kinetic models, it is evident that the fractional-order adsorption kinetic model is the best-suited approach for describing the CO_2_ adsorption capacity and reaction time. This model provides a more thorough and accurate description of adsorption phenomena that deviate from integer order kinetics (Fig. 14). It considers various factors, such as surface heterogeneity, multilayer adsorption, and the interactions between adsorbate molecules, which are all crucial in the intricate nature of the adsorption process^72^. Correlation coefficient values (R^2^) ranging from 0.97470 to 0.99337 at 5 bar (298, 308 K) suggest that the fractional-order adsorption kinetic model provides the best fit. Table 11 displays the corresponding kinetic parameters.

### Thermodynamic modeling

The thermodynamic parameters, including Gibbs free energy (ΔG°), Enthalpy (ΔH°), and Entropy (ΔS°), are essential for understanding the adsorption process^21^. Equations (13) provide a way to determine the values of ΔH° and ΔS° by plotting ln (K_L_) against the inverse of temperature (1/T) and can be used to calculate ΔG°. The universal gas constant (R) and the absolute temperature (T) are represented by 8.314 J/mol K and K, respectively.

13 $$\Delta {G}^{0}=-RT ln{K}_{d}$$

14 $$\Delta {G}^{0}=\Delta {H}^{0}-T\Delta {S}^{0}$$

15 $${K}_{d}=\frac{({P}_{i}-{P}_{f})V}{W}$$

16 $$ln{K}_{d}=\frac{\Delta {S}^{0}}{R}-\frac{\Delta {H}^{0}}{RT}$$

**Figure 15:**
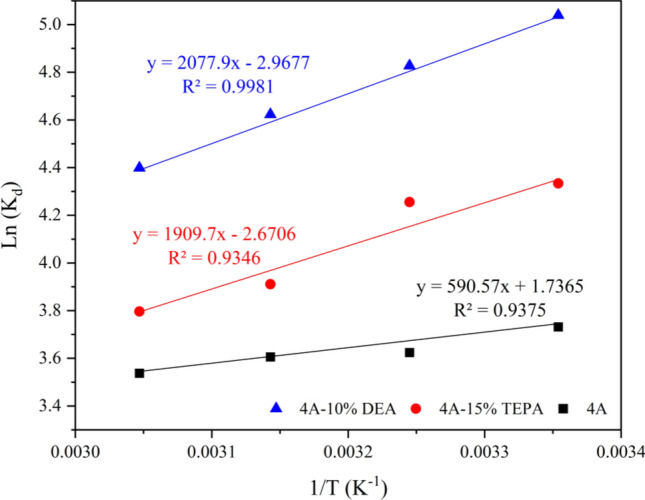
Arrhenius plot for the heat of adsorption.

Table 12 shows the thermodynamic parameters, with negative values of ΔG° that the spontaneity of the adsorption mechanism^23^. For 4A-zeolite, the ΔG° values shift inversely with temperature, indicating decreased adsorption feasibility at higher temperatures. The ΔG° values for 4A-zeolite are between − 9.219 and − 9.648 kJ/mol, suggesting physical adsorption. However, for DEA-4A and TEPA-4A, the ΔG° values increase with temperature, indicating both physical and chemical adsorption. By using (16, the values of ΔH° and ΔS° were obtained from the slope and intercept of ln (K) as a function of (1/T) (Fig. 15). ΔH° value for 4A-zeolite − 4.9, indicating an exothermic adsorption mechanism. Moreover, the ΔS° value suggests an associative mechanism for the adsorption process. Calculated thermodynamic data indicate the CO_2_ adsorption process on zeolite was physisorption, exothermic, and spontaneous^51^.

**Table 12:** Thermodynamic parameters for 4A-zeolite, 4A-15%TEPA and 4A-10%DEA.

Adsorbents	ΔH (kJ/mol)	ΔS (kJ/mol K)	ΔG (kJ/mol K)			
			298 K	308 K	318 K	328 K
4A	− 4.910	− 0.014	− 9.215	− 9.359	− 9.504	− 9.648
4A-15%TEPA	− 15.878	− 0.022	− 9.258	− 9.036	− 8.814	− 8.591
4A-10%DEA	− 17.277	− 0.025	− 9.920	− 9.673	− 9.426	− 9.179

### Adsorption capacity comparison with recent adsorbents

This section involved a comparative analysis between the current study and other relevant investigations focused on CO_2_ adsorption, employing various recent adsorbents. The outcomes of various similar studies were consolidated and presented in Table 13. Both 4A-10%DEA and the 4A-15%TEPA samples demonstrated considerable adsorption capacities, measuring 413.69 mg/g and 404.89 mg/g, respectively. A comparison between this study and the other research endeavors revealed the superior performance and a notable adsorption capability of the resulting amine-functionalized samples for CO_2_ capture applications.

**Table 13:** Some studies on CO_2_ capture by most recent adsorbents.

Sample	P (bar)	T (K)	q (mg/g)	Ref.
Ni-MOF-74	0.7	298	228.852	^73^
(MIL-53)	20	304	330.075	^74^
MOF-177-TEPA-20%	1	298	168.11	^31^
Amino-MIL-53	1	298	78	^32^
Commercial activated carbon	1	303	96.822	^75^
Activated carbon produced from shell	1	273	338.877	^76^
Molecular sieve	1	273	216.53	^77^
N-doped microalgae	1	273	293.99	^76^
Amine functionalized HCP (hypercrosslinked polymer)	9	298	441.18	^33^
Beta-25-APTMS	1	308	206.847	^64^
Zeolite Y-TEPA	1	303	114.426	^52^
Clinoptilolite-PF_6_	5	298	242.055	^6^
Zeolite 13X-Li^+^	1	298	307.19	^78^
Zeolite 4A	45	298	116.67	^28^
Zeolite 4A	5	298	325.374	This work
Zeolite 4A-15%TEPA	5	298	404.892	This work
Zeolite 4A-10%DEA	5	298	413.694	This work

### Adsorption mechanism

**Figure 16:**
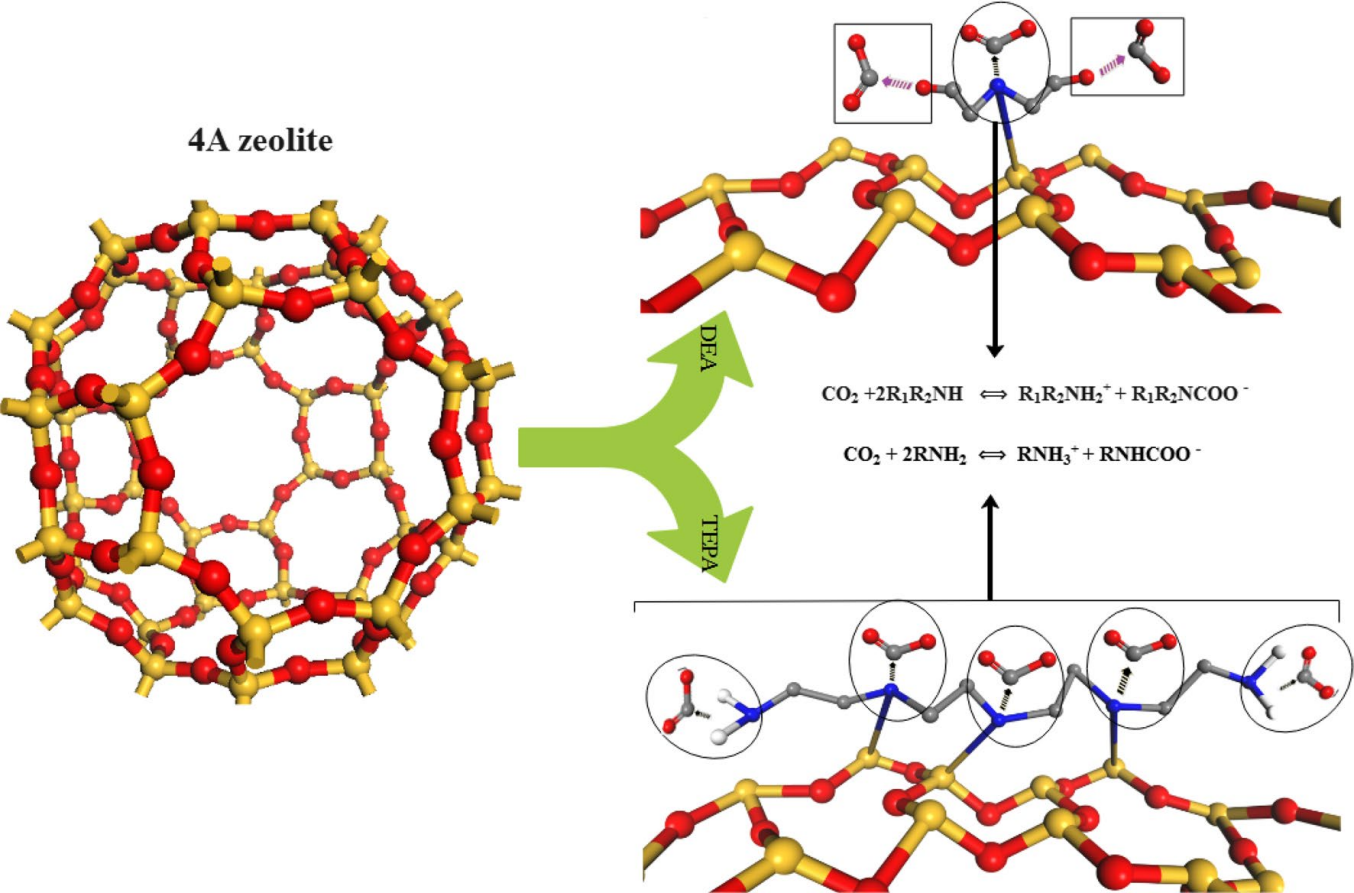
Schematic of CO_2_ adsorption on modified zeolites.

The adsorption mechanism of CO_2_ on amine-modified 4A-zeolite involves the chemisorption of CO_2_ molecules onto the amine groups present on the surface of the zeolite. The TEPA molecule comprises both primary amine (R_1_NH_2_) and secondary amine (R_1_R_2_NH) functional groups, both of which can participate in the reaction with CO_2_ and yield a carbamate ion, as shown in Fig. 16. On the other hand, the DEA molecule contains the R_1_R_2_NH functional group, which is secondary amine and is responsible for the chemical reaction between the amines and CO_2_. The presence of amine groups enhances the adsorption capacity of the material for CO_2_, while the increased hydrophilicity of the modified zeolite surface promotes the physisorption of CO_2_ molecules. The van der Waals forces between the zeolite and CO_2_ molecules, influenced by the Si and Al atoms in the zeolite structure, also play a role in the adsorption mechanism by attracting and holding CO_2_ molecules on the surface^49^. The strength of the interaction between CO_2_ molecules and the amine groups on the zeolite surface influenced factors such as the type of amine used for modification, the amine loading, and the pore size of the zeolite. Efficient capture of CO_2_ by amine-modified zeolites attributed to a combination of chemisorption and physisorption mechanisms.

### Cyclic performance

**Figure 17:**
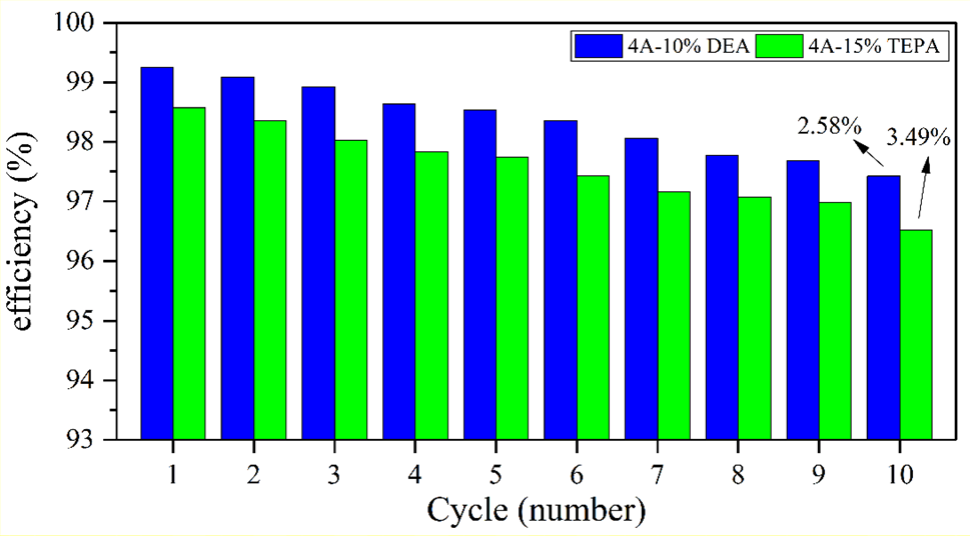
Adsorption–desorption of CO_2_ and reusability of amine-modified zeolites.

The ability to reuse the adsorbent is essential for industrial applications. Amine-based adsorbents may degrade or leach amines under harsh conditions, potentially affecting their long-term performance and reusability. In a series of ten adsorption cycles at 298 K and 5 bar, both types showed a slight decrease in adsorption potential after recycling at 410 K for 8 h. The 4A-15%TEPA adsorption potential was reduced by 3%, and the 4A-10%DEA adsorption potential decreased by approximately 2% (Fig. 17). These results suggest potential effectiveness in high-value industrial applications.

## Conclusion

We successfully synthesized and modified 4A-zeolite from kaolin to improve its performance in capturing CO_2_ in this study. To characterize the synthesized samples, we utilized several analytical techniques, including scanning electron microscopy (SEM), X-ray diffraction (XRD), Fourier-transform infrared spectroscopy (FT-IR), and Brunauer–Emmett–Teller (BET) testing. Our study assessed the effectiveness of modifications in enhancing the CO_2_ adsorption capacity of 4A-zeolite and tested the adsorption capacity of the modified zeolites at different temperatures and pressures. Utilizing Response Surface Methodology (RSM), we evaluated the CO_2_ adsorption performance of the modified zeolites by optimizing the operating conditions. CO_2_ adsorption experiments were performed at varying temperatures, pressures, and amine concentrations. The optimal adsorption capacity of the 4A-TEPA adsorbent is 477.342 mg/g, achieved at a temperature of 25.05 °C, pressure of 8.991 bar, and amine concentration of 15.275 wt%. Similarly, the 4A-DEA adsorbent exhibits an optimal adsorption capacity of 579.468 mg/g, with optimal operational variables of 25.270 °C, 8.870 bar, and 11.112 wt% amine concentration. After subjecting the adsorbents to recycling in an oven at 410 K for 8 h, the 4A-15%TEPA adsorption potential experienced a reduction of 3%, while the 4A-10%DEA adsorption potential showed a decrease of approximately 2%. The high R^2^ value of 0.9212 confirmed the excellent agreement between the experimental data and the model employed in this study. Furthermore, the kinetic and thermodynamic analyses have shown that the adsorption process of the modified zeolites is affected by both physisorption and chemisorption mechanisms. After analyzing various kinetic models, it was determined that the fractional-order adsorption model was the most appropriate. Overall, the results of this research highlight the promising potential of amine-functionalized 4A-zeolite as an effective adsorbent for CO_2_ capture. The material demonstrates notable advantages such as cost-effectiveness, high CO_2_ adsorption capacity, and a lack of reagent requirements. The method proposed in this study has the potential to facilitate the production of high-performance zeolites for various industrial applications.

## Supplementary Information

Supplementary Information.

## Data Availability

The datasets used and analyzed during the current study are available from the corresponding author upon reasonable request.
