# Supplementary material for: Improving the efficiency of 4A-zeolite synthesized from kaolin by amine functionalization for CO2 capture
Source: Sci Rep. 2023 Aug 2;13:12533. doi: 10.1038/s41598-023-39859-z (PMC10397218; doi:10.1038/s41598-023-39859-z)
Supplement: Supplementary file 1 — Supplementary Information. [file 41598_2023_39859_MOESM1_ESM.docx]

# Nomenclature

A radius of

ε represents the residual error term

ω Polanyi potential (J mol−1)

λ (D–R) model constant (mol2 J−2)

β polynomial coefficient

B first virial coefficient

f subscript that exhibits final condition

i subscript that exhibits initial condition

K_d_ distribution coefficient

K_F_ Freundlich model constant (mmol/g.bar)

K_L_ Langmuir model constant (1/bar)

MSE Mean Square Error

Mw Molecular weight (g/mol)

P pressure (bar)

Pc critical pressure

Pe equilibrium pressure (bar)

q adsorption capacity (mmol/g)

q_e_ equilibrium adsorption capacity (mmol/g)

q_m_ maximum adsorption capacity (mmol/g)

R gas constant (8.314 J/mol.K)

R^2^ correlation coefficient

S selectivity

SSR the sum of squares of regression

SSE the sum of squared errors

T temperature (^o^ C)

t time (min)

V reactor's volume

W mass of adsorbed gas (milligram)

w mass of adsorbent (g)

X RSM variable

Y RSM variable

Yi response value

($\hat{\boldsymbol{Y}_{\boldsymbol{i}}}$) response value obtained from the

($\bar{\boldsymbol{Y}_{\boldsymbol{i}}}$) mean of the responses

Z compressibility coefficient

ΔH Enthalpy change

ΔS Entropy change

ΔG Gibbs free energy change

**Table S1 : Experimental runs for CO_2_ adsorption**

|  |  | Factor 1 | Factor 2 | Factor 3 | Factor 4 | Response 1 | Response 2 |
| --- | --- | --- | --- | --- | --- | --- | --- |
| Std | Run | A:T | B:p | C:% loading | D:D | q | %adsorption |
|  |  | deg C | bar | % |  | mg/g | % |
| 21 | 1 | 45 | 5 | 15 | TEPA | 245.221 | 6.38158 |
| 23 | 2 | 45 | 5 | 15 | TEPA | 245.221 | 6.38158 |
| 45 | 3 | 45 | 5 | 15 | DEA | 229.85 | 5.71 |
| 13 | 4 | 45 | 5 | 5 | TEPA | 245.221 | 6.38158 |
| 1 | 5 | 35 | 3 | 10 | TEPA | 169.16 | 7.07915 |
| 46 | 6 | 45 | 5 | 15 | DEA | 229.85 | 5.71 |
| 9 | 7 | 25 | 5 | 15 | TEPA | 215.939 | 7.61321 |
| 38 | 8 | 45 | 9 | 15 | DEA | 416.9 | 5.79 |
| 17 | 9 | 45 | 5 | 15 | TEPA | 245.221 | 6.38158 |
| 8 | 10 | 55 | 7 | 20 | TEPA | 372.483 | 6.57806 |
| 18 | 11 | 45 | 5 | 15 | TEPA | 245.221 | 6.38158 |
| 31 | 12 | 35 | 3 | 20 | DEA | 143.85 | 5.98 |
| 2 | 13 | 55 | 3 | 10 | TEPA | 175.443 | 7.45296 |
| 3 | 14 | 35 | 7 | 10 | TEPA | 352.247 | 9.11195 |
| 4 | 15 | 55 | 7 | 10 | TEPA | 331.546 | 7.5 |
| 19 | 16 | 45 | 5 | 15 | TEPA | 245.221 | 6.38158 |
| 42 | 17 | 45 | 5 | 15 | DEA | 229.85 | 5.71 |
| 22 | 18 | 45 | 5 | 15 | TEPA | 245.221 | 6.38158 |
| 20 | 19 | 45 | 5 | 15 | TEPA | 245.221 | 6.38158 |
| 41 | 20 | 45 | 5 | 15 | DEA | 229.85 | 5.71 |
| 37 | 21 | 45 | 1 | 15 | DEA | 79.42 | 10.32 |
| 11 | 22 | 45 | 1 | 15 | TEPA | 85.9503 | 10.1823 |
| 34 | 23 | 55 | 7 | 20 | DEA | 350.33 | 6.38 |
| 10 | 24 | 65 | 5 | 15 | TEPA | 253.665 | 6.41199 |
| 25 | 25 | 45 | 5 | 15 | TEPA | 245.221 | 6.38158 |
| 14 | 26 | 45 | 5 | 25 | TEPA | 256.67 | 6.76163 |
| 50 | 27 | 45 | 5 | 15 | DEA | 229.85 | 5.71 |
| 51 | 28 | 45 | 5 | 15 | DEA | 229.85 | 5.71 |
| 24 | 29 | 45 | 5 | 15 | TEPA | 245.221 | 6.41199 |
| 6 | 30 | 55 | 3 | 20 | TEPA | 175.287 | 7.81944 |
| 5 | 31 | 35 | 3 | 20 | TEPA | 174.966 | 6.98752 |
| 30 | 32 | 55 | 7 | 10 | DEA | 362.56 | 6.58 |
| 27 | 33 | 35 | 3 | 10 | DEA | 150.29 | 6.1 |
| 47 | 34 | 45 | 5 | 15 | DEA | 229.85 | 5.71 |
| 48 | 35 | 45 | 5 | 15 | DEA | 229.85 | 5.71 |
| 36 | 36 | 65 | 5 | 15 | DEA | 204.15 | 5.38 |
| 44 | 37 | 45 | 5 | 15 | DEA | 229.85 | 5.71 |
| 40 | 38 | 45 | 5 | 25 | DEA | 216.08 | 5.3 |
| 15 | 39 | 45 | 5 | 15 | TEPA | 245.221 | 6.41199 |
| 29 | 40 | 35 | 7 | 10 | DEA | 386.56 | 6.68 |
| 32 | 41 | 55 | 3 | 20 | DEA | 140.17 | 6.02 |
| 12 | 42 | 45 | 9 | 15 | TEPA | 398.385 | 5.77361 |
| 52 | 43 | 45 | 5 | 15 | DEA | 229.85 | 5.71 |
| 39 | 44 | 45 | 5 | 5 | DEA | 212.28 | 5.2 |
| 35 | 45 | 25 | 5 | 15 | DEA | 412.99 | 9.48 |
| 7 | 46 | 35 | 7 | 20 | TEPA | 424.029 | 7.21204 |
| 28 | 47 | 55 | 3 | 10 | DEA | 155.06 | 7.07 |
| 16 | 48 | 45 | 5 | 15 | TEPA | 245.221 | 6.41199 |
| 26 | 49 | 45 | 5 | 15 | TEPA | 245.221 | 6.41199 |
| 49 | 50 | 45 | 5 | 15 | DEA | 229.85 | 5.71 |
| 43 | 51 | 45 | 5 | 15 | DEA | 229.85 | 5.71 |
| 33 | 52 | 35 | 7 | 20 | DEA | 402.39 | 6.92 |
